# Population genomic analysis reveals geographic structure and climatic diversification for *Macrophomina phaseolina* isolated from soybean and dry bean across the United States, Puerto Rico, and Colombia

**DOI:** 10.3389/fgene.2023.1103969

**Published:** 2023-06-07

**Authors:** Viviana Ortiz, Hao-Xun Chang, Hyunkyu Sang, Janette Jacobs, Dean K. Malvick, Richard Baird, Febina M. Mathew, Consuelo Estévez de Jensen, Kiersten A. Wise, Gloria M. Mosquera, Martin I. Chilvers

**Affiliations:** ^1^ Department of Plant, Soil and Microbial Sciences, College of Agriculture and Natural Resources, Michigan State University, East Lansing, MI, United States; ^2^ Ecology, Evolution and Behavior Program, Michigan State University, East Lansing, MI, United States; ^3^ Department of Plant Pathology and Microbiology, National Taiwan University, Taipei, Taiwan; ^4^ Department of Integrative Food, Bioscience and Biotechnology, Chonnam National University, Gwangju, Republic of Korea; ^5^ Department of Plant Pathology, University of Minnesota, St. Paul, MN, United States; ^6^ BCH-EPP Department, Mississippi State University, Mississippi State, MS, United States; ^7^ Department of Plant Pathology, North Dakota State University, Fargo, ND, United States; ^8^ Department of Agroenvironmental Sciences, University of Puerto Rico, Mayagüez, PR, United States; ^9^ Department of Plant Pathology, College of Agriculture, Food and Environment, University of Kentucky, Princeton, KY, United States; ^10^ Plant Pathology, Crops for Nutrition and Health, International Center for Tropical Agriculture (CIAT), The Americas Hub, Palmira, Colombia

**Keywords:** charcoal rot, climate change, genotype–environment associations, pathogen adaptation, redundancy analysis, phylogenomics, landscape genetics

## Abstract

*Macrophomina phaseolina* causes charcoal rot, which can significantly reduce yield and seed quality of soybean and dry bean resulting from primarily environmental stressors. Although charcoal rot has been recognized as a warm climate-driven disease of increasing concern under global climate change, knowledge regarding population genetics and climatic variables contributing to the genetic diversity of *M. phaseolina* is limited*.* This study conducted genome sequencing for 95 *M. phaseolina* isolates from soybean and dry bean across the continental United States, Puerto Rico, and Colombia. Inference on the population structure using 76,981 single nucleotide polymorphisms (SNPs) revealed that the isolates exhibited a discrete genetic clustering at the continental level and a continuous genetic differentiation regionally. A majority of isolates from the United States (96%) grouped in a clade with a predominantly clonal genetic structure, while 88% of Puerto Rican and Colombian isolates from dry bean were assigned to a separate clade with higher genetic diversity. A redundancy analysis (RDA) was used to estimate the contributions of climate and spatial structure to genomic variation (11,421 unlinked SNPs). Climate significantly contributed to genomic variation at a continental level with temperature seasonality explaining the most variation while precipitation of warmest quarter explaining the most when spatial structure was accounted for. The loci significantly associated with multivariate climate were found closely to the genes related to fungal stress responses, including transmembrane transport, glycoside hydrolase activity and a heat-shock protein, which may mediate climatic adaptation for *M. phaseolina*. On the contrary, limited genome-wide differentiation among populations by hosts was observed. These findings highlight the importance of population genetics and identify candidate genes of *M. phaseolina* that can be used to elucidate the molecular mechanisms that underly climatic adaptation to the changing climate.

## 1 Introduction

Understanding the genetic diversity of plant pathogen populations and the factors influencing it allow for inferences about their evolutionary potential and identification of the molecular bases of adaptation. Plant pathogens are often genetically structured in different agricultural landscapes as a result of geographic and environmental differences (Gladieux et al., 2014; McDonald and Stukenbrock, 2016). Among different environments, agroecosystems provide remarkable conditions for rapid adaptation of plant-pathogenic fungi due to various abiotic and biotic factors such as genetic crop uniformity of monocultures, the prevalent occurrence of human-mediated migration (Wingfield et al., 2015; Crous et al., 2017), and intrinsic characteristics of fungi such as their mode of reproduction (McDonald and Stukenbrock, 2016). These factors are strong drivers of genomic divergence and adaptation in plant pathogenic fungi (Stukenbrock et al., 2011; Savolainen et al., 2013; Croll and Mcdonald, 2016). However, characterizing how selective pressures of abiotic and biotic factors contribute to population genetics of plant-pathogenic fungi remains challenging.

*Macrophomina phaseolina* is a seed- and soil-borne fungal pathogen that infects more than 500 host species (Batista et al., 2021), and causes dry root rot, seedling blight, crown rot and charcoal rot in many important economic and subsistence crops worldwide, including soybean (*Glycine max*) and dry bean (*Phaseolus vulgaris*) (Dhingra and Sinclair, 1978; Yang and Navi, 2005; Saleh et al., 2010; Jacobs et al., 2019). During host infection, *M. phaseolina* invades the xylem preventing water uptake, causing wilting and premature plant death (Mengistu et al., 2007; Romero Luna et al., 2017). These symptoms can develop rapidly causing extensive yield loss and grain or seed quality reduction (Smith and Carvil, 1997). Charcoal rot of soybean has been ranked seventh out of 25 pests and pathogens causing global yield losses (Savary et al., 2019), with the potential for yield reductions within individual fields of up to 50% (Wrather et al., 2001). In the United States, charcoal rot ranked among the top seven most destructive diseases with economic losses totaling 220 billion dollars from 2010 to 2014 (Allen et al., 2017). Disease development is favored by hot and dry conditions (Dhingra and Sinclair, 1974), with colonization in the soybean and dry bean tap root and lower stem being greatest under high temperatures (28°C—35°C) and low soil moisture (Dhingra and Sinclair, 1974; Meyer and Sinclair, 1974; Kendig et al., 2000; Mengistu et al., 2011; Reznikov et al., 2018).

*Macrophomina phaseolina* is haploid, reproduces asexually, and overwinters in soil and crop residue as abundant, melanized microsclerotia that serve as the primary inoculum to initiate infection in subsequent seasons (Gupta et al., 2012; Islam et al., 2012). Pycnidia are occasionally produced on soybean and other host plants, however, their epidemiological significance has yet to be fully defined (Knox-Davies, 1965; Dhingra and Sinclair, 1978; Mihail and Taylor, 1995). Depending on environmental conditions*, M. phaseolina* may survive as microsclerotia in soil for up to 15 years (Short et al., 1980; Baird et al., 2003), and for up to 3 years as microsclerotia in symptomatic seeds or as mycelium in asymptomatic seeds (Hartman et al., 2015). To date, no clonal lineages or pathotypes have been identified for *M. phaseolina*, despite reports of within-species variation in morphology and pathogenicity (Dhingra and Sinclair, 1973; Dhingra and Sinclair, 1978; Sexton et al., 2016). Population genetic studies based on microsatellite markers of isolates representing different geographic regions and hosts across the United States have found moderate to high genetic diversity and mixed evidence of population structure by host or geography. Although considerable efforts have been focused on ascertaining host specialization, it is generally concluded that there is no strong evidence of this specificity, in which isolates from one plant species can often cause disease in other plant species (Su et al., 2001; Zveibil et al., 2012; Romero Luna et al., 2017). Nevertheless, genetic similarity of isolates according to host and United States regions and some degree of host preference have been noted (Su et al., 2001; Jana et al., 2005; Baird et al., 2010; Saleh et al., 2010; Arias et al., 2011). Notably, a group of *M. phaseolina* isolates obtained from strawberry in California were found to form a species-specific cluster, exhibiting strong host preference for strawberry over other hosts around California (Koike et al., 2016; Burkhardt et al., 2019).

Studying population genetics using statistical methods that leverage genomic, geographic and environmental data can account for continuous and discrete genetic variation and provide insights into the genetic basis underlying environmental adaptation (Hoban et al., 2016; Bontrager and Angert, 2018; Bradburd et al., 2018). These approaches may be used to identify environmental factors driving selection and provide an understanding of how and why pathogen populations vary across space. Population genomics and genotype-environment associations have been applied in numerous studies to resolve the basis of rapid adaptation and identify candidate adaptive loci associated with environmental variation (Lasky et al., 2012; Forester et al., 2018; Xuereb et al., 2018; Gibson and Moyle, 2020; Capblancq and Forester, 2021). However, characterizing population structure and unravelling the effects of continuous or discrete processes on the genetic differentiation remains challenging for many plant-pathogenic fungi.

A major challenge arises because continuous geographic differentiation (e.g., isolation by distance or climatic variation along a gradient) can be confounded with discrete processes such as admixture and long-distance migration (human-mediated migration) which are commonly observed in plant pathogens (Wingfield et al., 2015; Crous et al., 2017; Tabima et al., 2019; LeBlanc et al., 2021). In addition, collinearity between spatial and environmental variables makes it difficult to elucidate to what extent geographic and environmental differences may be contributing to genetic differentiation. To address these issues, multivariate statistical methods, specifically redundancy analysis (RDA), have been increasingly used to disentangle the effects of environmental factors in shaping genetic variation. RDA is a type of constrained ordination in which a set of SNPs are modeled as responses in a function of combinations of environmental predictors. Because of its ability to evaluate many loci simultaneously, RDA has been found to be superior to traditional mixed-models associations methods in identifying weak, multilocus selection (Forester et al., 2018), suggestive of polygenic adaptation. Furthermore, partial RDA models, in which covariables can be included, has been used to account for underlying population structure in the identification of loci associated with environmental factors for climate adaptation in a variety of systems including plant and animal species (Lasky et al., 2012; Forester et al., 2018; Xuereb et al., 2018; Gibson and Moyle, 2020; Capblancq and Forester, 2021).

Climate fluctuation and temperature in particular are important abiotic factors leading to local adaptation of plant-associated fungi (Savolainen et al., 2013; Croll and Mcdonald, 2016), especially in species occupying spatially and climatically heterogeneous environments (Ellison et al., 2011; Branco et al., 2015; Branco et al., 2016; Fitzpatrick and Keller, 2015). *Macrophomina phaseolina* is recognized for its different ecological roles as an endophyte, saprotroph, and latent or opportunistic pathogen with broad geographic distribution (Dhingra and Sinclair, 1974; Slippers and Wingfield, 2007; Slippers et al., 2013; Parsa et al., 2016; Crous et al., 2017). Worldwide diseases caused by *M. phaseolina* have re-emerged in recent decades, with outbreaks occurring in tropical and subtropical regions as well as temperate regions (Leyva-Mir et al., 2015; Casano et al., 2018; Koehler and Shew, 2018; Meena et al., 2018; Nishad et al., 2018; Tančić Živanov et al., 2018; Wang et al., 2020). In the United States, charcoal rot of soybean has been a primary issue in southern and central states historically. However, charcoal rot has been noted to occur in northern states such as Wisconsin, New York, Minnesota, and Michigan (Yang and Navi, 2005; Bradley and del Río, 2007; ElAraby et al., 2007; Baird et al., 2010; Cummings and Bergstrom, 2013) and currently it is a consistent threat to soybean production across southern and northern states (Bradley et al., 2021; Roth et al., 2021). Although many factors may influence disease incidence, greater disease and yield losses have been observed in years with high temperature and low soil moisture (Bradley and Allen, 2014; Allen et al., 2017). A recent study concluded that *M. phaseolina* isolates were regionally adapted when comparing isolates from the northern and southern United States states (Sexton et al., 2016). Investigations in the context of species within the Botryosphaeriaceae family suggest that geographical distribution and host affinity dynamics in *M. phaseolina* are strongly influenced by climate due to its broad host range and ecologically diverse roles (Slippers and Wingfield, 2007; Batista et al., 2021), while recent reviews have indicated that the impact of charcoal rot on crop losses may intensify in the face of global warming (Basandrai et al., 2021; Pandey and Basandrai, 2021; Cohen et al., 2022). These factors, together with predicted increases in temperature and extreme rainfall variation as projected in the climatic change models (IPCC, 2022), make it critical to better understand genetic structure and climatic factors as potential selection agents of *M. phaseolina*.

The broad geographic distribution and population dynamics of *M. phaseolina* suggest that populations in the continental United States, Puerto Rico and Colombia might have been influenced by a complex environmental and agricultural landscape and may be structured and differentially adapted at a continental or regional level. In the present study, the first aim was to better understand the genetic structure in *M. phaseolina* populations isolated from soybean and dry bean across the United States, Puerto Rico and Colombia using genome-wide single nucleotide polymorphisms (SNPs). Specifically, the contribution of discrete vs continuous genetic differentiation was assessed by inferring *M. phaseolina* genetic groups while accounting for geographic isolation by distance. The hypotheses tested were *M. phaseolina* populations differentiated i) between countries and ii) between hosts within the United States The second aim was to investigate whether climatic variables contribute to patterns of adaptive genetic variation in *M. phaseolina*. Using RDA, the hypotheses were i) specific climatic variables contribute to genetic variation, ii) climatic variables independently contribute to patterns of genetic variation when accounting for underlying spatial and population structure, and iii) loci in strong association with multivariate climate can be identified and have roles in driving local adaptation to climate.

## 2 Results

### 2.1 Whole-genome sequencing for 95 *Macrophomina phaseolina* isolates

**FIGURE 1:**
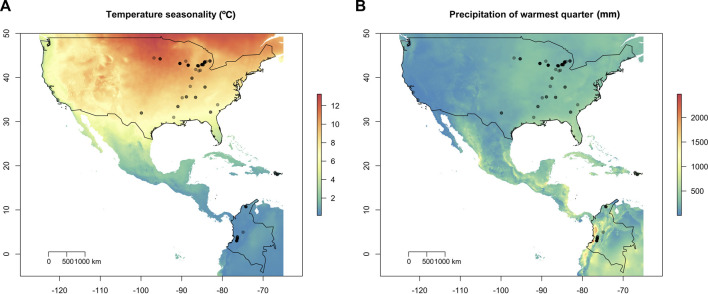
Geographic location of the 95 *Macrophomina phaseolina* isolates overlaid on temperature and precipitation variables. **(A)** Isolate collection sites overlaid on temperature seasonality (standard deviation; °C). Temperature seasonality contributed the most to explaining patterns of spatial genetic variation using redundancy analysis (RDA). **(B)** Isolates overlain on precipitation warmest quarter (mm). United States, Puerto Rico and Colombia are outlined in black.

Whole-genome resequencing was completed for 95 *M. phaseolina* isolates collected across the United States, Puerto Rico, and Colombia, including 52 soybean isolates, 40 dry bean isolates, two strawberry isolates, and one Ethiopian mustard isolate (Figure 1; Supplementary Table S1). Sequence coverage varied across individual isolates from 5X to 85X, across 93% of the Macpha1 reference genome (JGI Mycocosm, MPI-SDFR-AT-0080 v1.0). A total of 2.8 million SNPs were identified across all isolates, and a mean read depth (DP) of 12X was obtained for all SNPs after filtering. Most SNPs had a mapping quality (MQ) value equal to 60 (94%) and SNPs with MQ values <60 were removed. The distribution of missing data across the isolates and across the variants was even, with most individuals representing similar missing data (0%–0.006%), and all variants containing missing data were removed. The final data set contained 76,981 high-quality biallelic SNPs in all isolates, and the data set was retained for all analyses.

### 2.2 Phylogenomics differentiated 95 isolates into two main clades of the United States and Colombian-Puerto Rican origins

**FIGURE 2:**
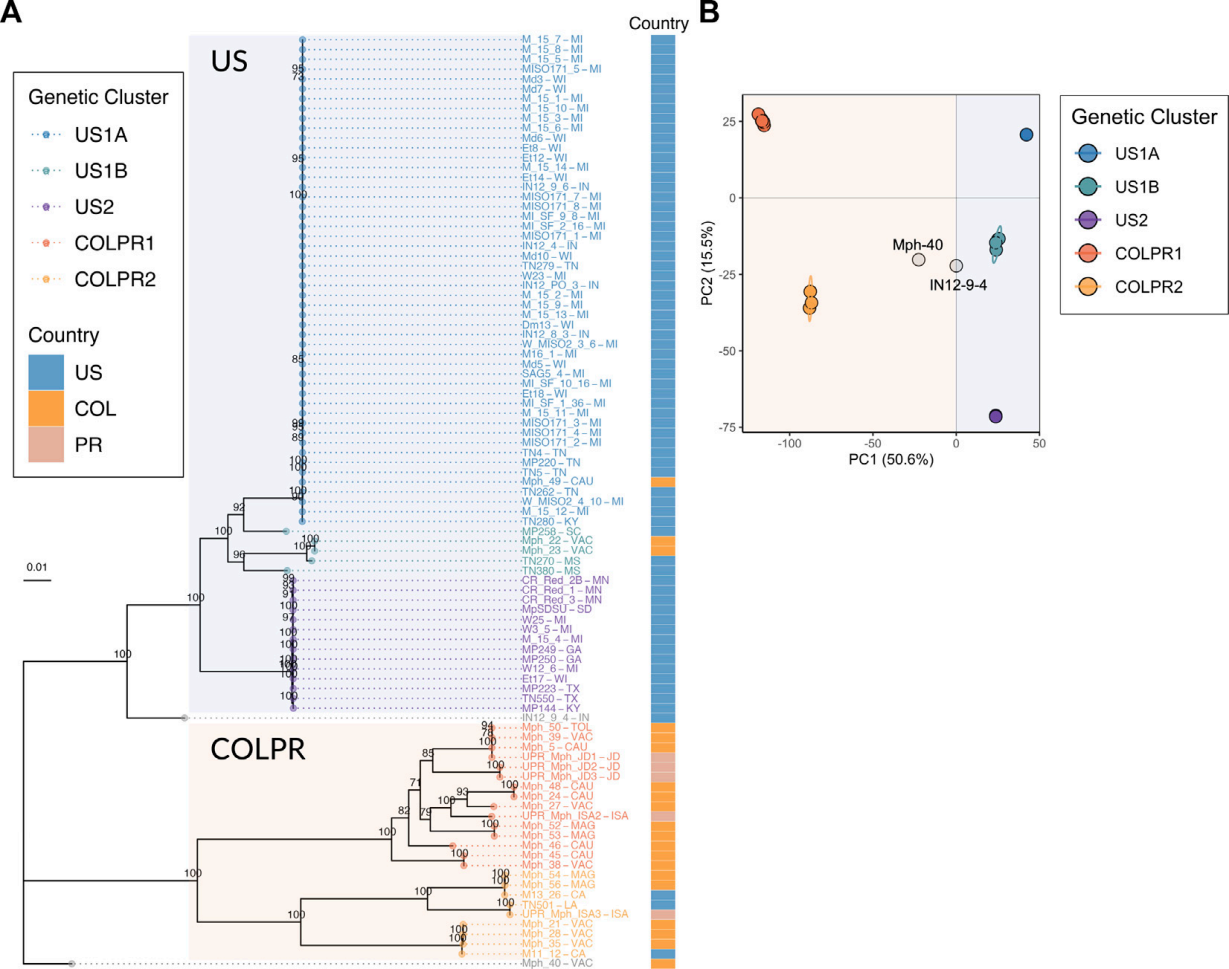
Population structure of *Macrophomina phaseolina* in the United States, Colombia and Puerto Rico reveals five genetic clusters in a pattern of hierarchical structure. **(A)** Maximum-likelihood phylogeny reconstructed using 76,981 high-quality SNPs. Bootstrap support values over 70 are shown at nodes. Bootstrapping converged after 400 replicates. Colored tips represent the genetic cluster for each isolate as defined by principal components analysis. The two main clades, US and COLPR, are highlighted by rectangular shading. The country of collection for each isolate is denoted by colored squares at the right bar. **(B)** Scatterplot from a principal component analysis based on the two first PCs (the eigenvectors of the SNP dataset) for all isolates. Points are colored by membership in the five genetic clusters. Isolate names include states/municipalities codes: CA: California, CAU: Cauca, GA: Georgia, IN: Indiana, ISA: Isabela, JD: Juana Diaz, KY: Kentucky, LA: Louisiana, MAG: Magdalena, MI: Michigan, MN: Minnesota, MS: Mississippi, SC: South Carolina, SD: South Dakota, TN: Tennessee, TOL: Tolima, TX: Texas, VAC: Valle del Cauca, WI: Wisconsin. Country codes: US: United States, COL: Colombia and PR: Puerto Rico.

To infer the genetic similarity in *M. phaseolina* isolates across the continental United States, Colombia and Puerto Rico, a maximum-likelihood (ML) phylogenetic tree based on the 76,981 SNPs was constructed. Five genetic clusters were identified across the United States (n = 3), Colombia and Puerto Rico (n = 2). Furthermore, a pattern of hierarchical structure differentiating the United States and Colombian-Puerto Rican isolates was observed. The ML tree provided strong support (100% bootstrap) for two main clades, hereafter referred to as US and COLPR, and five well-supported clades within the main clades (Figure 2A). The United States isolates M11–12 and M13-26 from California, and TN501 from Louisiana clustered in the COLPR clade, while the Colombian isolates Mph-22, Mph-23, and Mph-49 fell within the US clade (Figure 2A). Other than these six isolates, all isolates from the United States were placed in the US clade, and all isolates from Colombia and Puerto Rico were grouped in the COLPR clade.

There were three subclades (US1A, US1B and US2) within the US clade and two subclades (COLPR1 and COLPR2) within the COLPR clade. The PCA clustered isolates in five distinct groups in agreement with phylogenetic analysis, with little evidence of within group differentiation (Figure 2B). The first PC explains most of the variance (50.6%) and separates out isolates in the US clade from the isolates in the COLPR clade, while the second PC explains 15.5% of the variance dividing isolates into the five groups in the phylogenetic analysis (Figure 2B). An exception was isolate MP258 which in the PCA was grouped in US1B instead of US1A. Since the phylogenetic and PCA clustering revealed essentially the same hierarchical groupings, they were named genetic clusters US1A, US1B, US2, COLPR1 and COLPR2.

US1A isolates represented the predominant group in the United States, with most isolates collected in the East North Central and Central regions in the states of Michigan (29), followed by Wisconsin (11), Indiana (5), Tennessee (5) and Kentucky (2). Cluster US1B was represented by isolates from Mississippi (2) and South Carolina (1). US2 isolates represented the second largest group in the United States and were mostly collected in the Northern Great Plains [Minnesota (3), South Dakota (1)] and South [Texas (2) and Georgia (2)] regions. Also, within this cluster were isolates from Wisconsin (1), Michigan (4), and Kentucky (1). In contrast, the COLPR1 cluster grouped most isolates from Colombia (11) and Puerto Rico (4) while COLPR2 grouped isolates from Colombia (5), one isolate from Puerto Rico, and three isolates from the United States. No evidence of population structure by states was found, which indicated that states do not represent genetic groups and *M. phaseolina* is genetically structured at a broader subcontinental regional extent.

A ML phylogeny rooted with the *M. phaseolina* Macpha1 reference genome was reconstructed using the set of high-quality SNPs. The Macpha1 reference genome was considered as a suitable outgroup based on its European and *Arabidopsis thaliana* origin. The phylogenetic reconstruction with Macpha1 as a root revealed the COLPR2 clade as an outgroup to all other clades, while the United States clades were reconstructed as terminal clades (Supplementary Figure S1). The topology of the rooted ML phylogeny indicated the COLPR clades as more diverse than the major US terminal clades (US1A and US2). This higher diversity in COLPR clades was indicated by longer average branch length than in the US clades, representing a higher average number of substitutions per site. Differences in diversity can also be inferred from the PCA clustering. In PC space, 50 isolates in US1A and 14 isolates in US2 genetic clusters clustered effectively on top of each other, while isolates in US1B, COLPR1 and COLPR2, although projected near each other, clustered distinctively more dispersed (Figure 2B). The placement of COLPR genetic clusters and their higher diversity as compared to US genetic clusters indicates them as potential sources to the US clusters.

To test the relatedness of *M. phaseolina* isolates from soybean and dry bean in United States, the host information was mapped to the ML tree (Supplementary Figure S1A). Generally, isolates that shared a common host did not cluster within genetic clusters in the United States. Isolates collected from soybean and dry bean grouped together in the two larger United States genetic clusters (US1A and US2; Supplementary Figure S1A). This lack of structure was further supported in a PCA showing overlapping ellipses representing 95% of the isolates from each of the hosts (Supplementary Figure S2).

### 2.3 Spatial population structure defines discrete population structure in *Macrophomina phaseolina* between the United States and Colombia-Puerto Rico and continuous substructure between genetic clusters within US and COLPR clades

To infer the number of distinct genetic groups in *M. phaseolina* while accounting for continuous geographic differentiation, spatial analysis of population structure was conducted using a Bayesian (conStruct) and a model-free matrix factorization (TESS3) framework. Spatial analysis of population structure incorporates geographic distance in the estimation of ancestry coefficients (the proportion of individual isolate’s genome originating from the ancestral genetic group, K). The genetic structure of the 95 isolates was explained better by a spatial model of admixture between discrete genetic groups, where isolation by distance was accounted for rather than the non-spatial model. This was indicated by the increase in predictive accuracy in the conStruct spatial models for all tested values of K (referred hereafter as layers in conStruct framework; Supplementary Figure S3B). This suggests that isolation by distance or climatic gradients likely play a role in shaping patterns of genetic variation in the sampled isolates.

**FIGURE 3:**
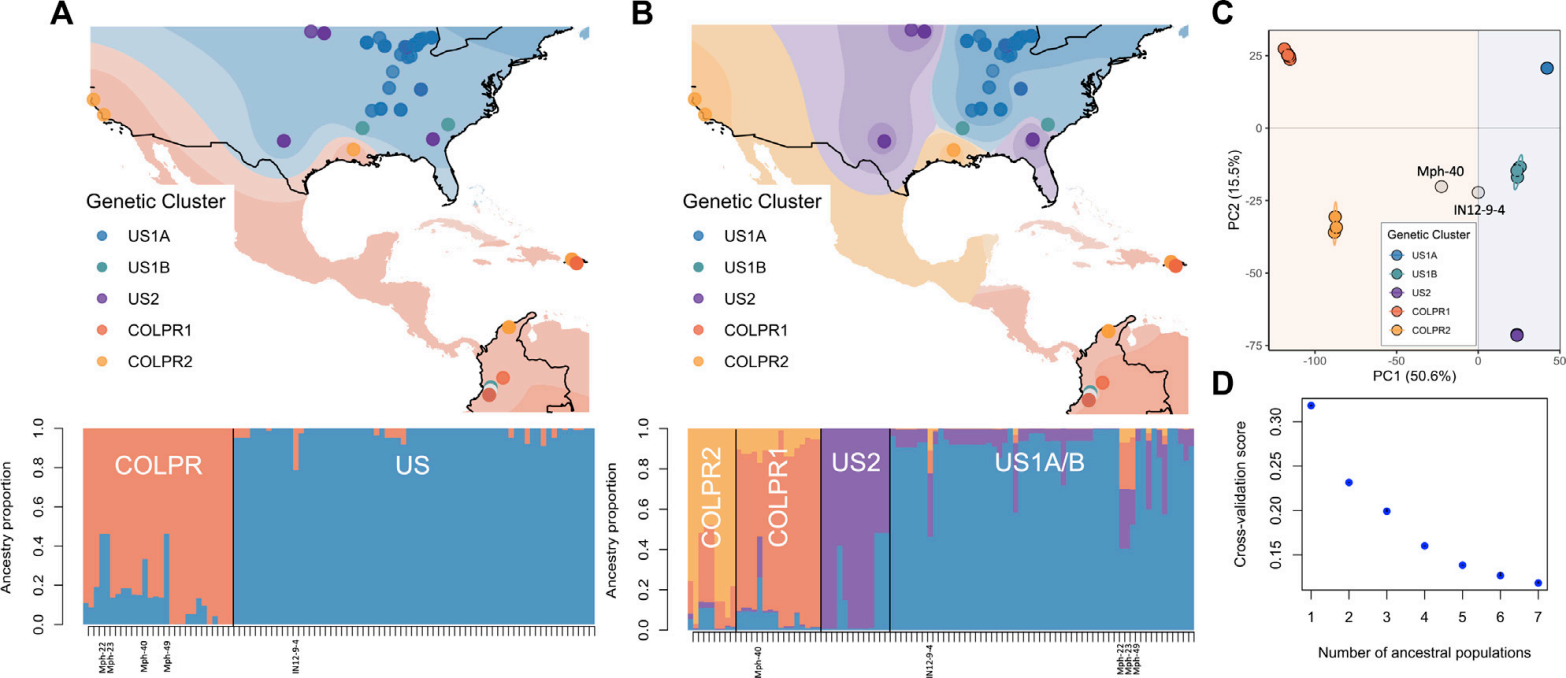
Spatial population structure defines discrete population structure in *Macrophomina phaseolina* between the United States and Colombia-Puerto Rico and continuous substructure between genetic clusters. **(A)** Isolate membership to ancestral populations identified with TESS3 using K = 2 and **(B)** K = 4. Top: Isolate collection sites overlaid on individual membership, each color representing a population. Each point represents an isolate, points are colored by their assignment to genetic clusters as identified in principal component analysis to show agreement between the methods. Bottom: Ancestry proportions of all isolates. Isolates identified as admixed (Mph-22, Mph-23, Mph-49, Mph40 and IN129-4) at K = 2 are labeled. **(C)** Scatterplot from a principal component analysis for all isolates (from Figure 2). **(D)** Values of the TESS3 cross-validation criterion (root mean-squared errors, RMSE) as a function of the number of ancestral populations (K = 1 to K = 7).

Spatial population structure description using TESS3 returned the greatest decrease in root mean-squared errors at K = 2 (0.087, from 0.318 at K = 1 to 0.232 at K = 2; Figure 3D) and detected the US and COLPR clades. At K = 2, TESS3 spatial estimation strongly assigned 95% of isolates to a single ancestral population (ancestry proportion Q > 0.8; Figure 3A). All isolates in the US clade, except for the three isolates collected in Colombia, were identified as being derived from a single ancestral population (represented by blue; Figure 3A, bottom). Likewise, all COLPR isolates are estimated to have a majority component of ancestry from a single source population (represented by orange; Figure 3A, bottom) including the three isolates collected in the United States (M11–12 and M13-26 from California, and TN501 from Louisiana). The three isolates collected in Colombia grouping in the United States clade (Mph-22, Mph-23 and Mph-49) were identified as admixed (i.e., to have ancestry from more than one population instead of drawing ancestry mostly [Q > 0.8] from a single ancestral population) between the two ancestral groups (Figure 3A, bottom) as well as the two isolates (IN12-9-4 from Indiana and Mph-40 from Colombia) placed outside the supported clusters in the ML tree and PCA. At K = 4, further substructure was detected that generally reflect the genetic clusters within the US and COLPR clades; except that an ancestral population for US1B isolates was not inferred (Figure 3B). The decrease in root mean-squared errors at K = 4 (0.04; from 0.20 at K = 3 to 0.16 at K = 4; Figure 3D) was the second largest value after that at K = 2, reflecting the hierarchical structure observed in previous analyses. However, although isolates in each genetic cluster (except US1B) were inferred as drawing the most ancestry from their own ancestral population, only 76% of isolates had an ancestry proportion Q) > 0.80 to a single ancestral population (Figure 3B, bottom), demonstrating weaker assignments than those at K = 2.

Consistently, the results from conStruct spatial model with K = 2 returned the greatest increase in predictive accuracy and primarily partitioned the isolates in two main groups mostly in line with US and COLPR clades (Supplementary Figure S3A). Based on cross-validation results, the predictive accuracy increased with increasing values of K (Supplementary Figure S3B), however additional layers beyond K = 2 contribute little to total covariance (Supplementary Figure S3C). Therefore, supporting two discrete ancestral populations while population substructure can be explained by continuous genetic differentiation.

Taken together conStruct and TESS3 results supported two discrete genetic groups for the US and COLPR main clades and suggested that most isolates within US and COLPR clades can be better described to have ancestry mainly from each single ancestral population. It may therefore be reasonable that the evolutionary processes leading to divergence between genetic clusters within the US (US1A, US1B, US2) and COLPR (COLPR1 COLPR2) clades were associated to isolation by distance or climatic differences rather than different discrete ancestry.

### 2.4 Genetic diversity and differentiation between the US and COLPR clades and genetic clusters of *Macrophomina phaseolina*

To examine genome-wide diversity of *M. phaseolina* within and among clades and genetic clusters, we estimated gene diversity (He) and median pairwise genetic distance for each of the clades and genetic clusters. Pairwise genetic distance showed that COLPR isolates had greater genetic distances among isolates than those in the US clade, with a gene diversity (He) significantly higher in the COLPR clade (0.236) than the US clade (0.068; Table 1) (Hs.test, *p* = 0.002). Among clusters, the COLPR2 cluster has the highest genetic diversity, considering both gene diversity and pairwise genetic distance, followed by COLPR1, US1B, US2, and the US1A cluster has the lowest values (Table 1). The higher genetic distance among isolates in the US1B cluster as compared to other US clusters, likely reflects that the cluster is only represented by five isolates of which two were collected in Mississippi, two in Colombia and one in South Carolina.

**TABLE 1:** Summary statistics for genetic diversity of *Macrophomina phaseolina* clades and genetic clusters. N is number of isolates (sample size); MLG is number of observed multilocus genotypes; eMLG is the number of expected MLG at a sample size of 25 for clades and 5 for genetic clusters based on rarefaction. MLL is number of observed multilocus lineages by population using a bitwise cutoff distance of 0.0001; CF is clonal fraction (1 - (MLL/N). Clone corrected values are shown and indicated by asterisks for indices of genotypic diversity: Shannon-Wiener Index (H*), Stoddart and Taylor’s Index (G*), Simpson’s index (lambda*) and evenness (E5*).

Clade, genetic cluster	N	Gene diversity (He)	Median pairwise genetic distance	MLG	eMLG	MLL	eMLL	CF	H*	G*	Lambda*	E5*
US	70	0.0680	0.0890	54	21.75	19	10.70	0.73	1.990	5.53	8	0.702
US1A	50	0.0001	0.0002	34	4.71	6	2.87	0.88	0.935	2.48	0.561	0.883
US1B	5	0.0540	0.0900	5	5	4	4	0.20	1.332	3.57	0.720	0.922
US2	14	0.0010	0.0008	14	5	8	4.26	0.43	1.4	3.99	0.738	0.950
COLPR	25	0.2360	0.3320	25	25	15	15	0.40	2.640	13.30	0.925	0.942
COLPR1	15	0.0840	0.0960	15	5	9	4.43	0.40	1.456	4.24	0.756	0.962
COLPR2	9	0.1510	0.2650	9	5	5	3.73	0.44	1.242	3.35	0.688	0.924

Note: Summary statistics were calculated using the clone-corrected data at 79 MLGs

To evaluate genotypic diversity both in terms of genotypic richness (the number of observed genotypes) and evenness of distribution of genotypes, the number of multilocus genotypes (MLG) was calculated for each clade and genetic cluster. A MLG was defined as a unique combination of SNPs. Given the large number of 76,981 SNPs and genotyping error rate from NGS data, it is unlikely that a true clone will be represented by an MLG. Thus, to better represent clones, closely related genotypes were collapsed into multilocus lineages (MLLs) based on a Prevosti’s genetic distance threshold of 0.0001 (8 SNPs). Of the 95 isolates, 79 had unique genotypes (MLGs) corresponding to 34 MLLs (Table 1). eMLG and eMLL are the number of expected MLGs and MLLs based on rarefaction at the lowest common sample size between clades and genetic clusters and were used to allow comparisons across them given their unequal sample sizes.

Genotypic richness was highest in the COLPR clade (15 eMLLs) as compared to the US clade (10.7 eMLLs). Among genetic clusters, the COLPR1 cluster had the highest number of eMLLs, followed by US2, US1B, COLPR2 and US1A. This indicates genotypic richness is highest in COLPR1 and lowest in the US1A genetic cluster, in which more than 80% of the isolates were clonal (Table 1, CF). Although, lower genotypic richness is inferred in COLPR2 and US1B as compared to the gene diversity pattern, this may be due to their low sample size. Evenness and the corrected Shannon-Wiener’s index, Stoddart and Taylor’s index and Simpson’s Index, were all highest in the COLPR clade than in the US clade and followed the same pattern among genetic clusters as with genotypic richness (Table 1). Finally, there were no shared MLGs or MLLs among genetic clusters.

Similarly, between countries, significantly higher gene diversity in Colombia (0.263) compared with the United States (0.104) (Hs.test, *p =* 0.002). Gene diversity in Puerto Rico (0.163) was intermediate and not significantly different from the United States (Hs.test, *p =* 0.218) or Colombia (Hs.test, *p =* 0.396). Pairwise genetic distances, corrected genotypic diversity indices and evenness calculated for each country follow the same pattern of gene diversity (Supplementary Table S3). To infer migration among countries by tracking genotype flow, MLLs shared among countries were identified. A total of three MLLs were shared among countries. The MLL with one isolate from Colombia (Mph-5) and one from Puerto Rico (UPR-Mph-JD1) clustering in COLPR1, the MLL with one isolate from Puerto Rico (UPR-Mph-ISA3) and one from Louisiana (TN501) clustering in COLPR2, and the MLL with one isolate from Colombia (Mph-49) and 19 isolates from the United States clustering in US1A (Supplementary Figure S4). In addition, all populations clustering approaches indicated that Colombian isolates Mph-22 and Mph-23 are the most closely related to the United States isolates clustered in US1B, and Californian isolates M13–26 and M11-12 are the most closely related to Colombian isolates clustering in COLPR2. The rooted ML tree indicated isolate Mph-40 (from Colombia) as an outgroup to US clusters and discriminatory analysis of principal components (DAPC) clustered this isolate along with IN12-9–4 (from Indiana) with US1B isolates (Supplementary Figure S1). Overall, migration between Colombia, Puerto Rico and United States is a likely scenario.

To test the hypothesis that genetic clusters of *M. phaseolina* are differentiated, we used hierarchical analysis of molecular variance (AMOVA) and Nei’s G_ST_ (an F_ST_-analogous genetic differentiation measure applicable to haploids). Populations were significantly differentiated among clades, genetic clusters, as well as within genetic clusters (*p* < 0.001; Supplementary Table S2). AMOVA revealed that most of the total genetic variance was partitioned among US and COLPR clades (47%) and among genetic clusters (42%), and only 11% within genetic clusters. Consistently, very high genetic differentiation was found between US and COLPR clades (G_ST_ = 0.45) and among genetic clusters (G_ST_ = 0.50–0.99; Table 2). The COLPR2 (G_ST_ = 0.50–0.69) and US1B (G_ST_ = 0.54–0.69) clusters had the lowest G_ST_ when compared with any other cluster. Differentiation was lowest between COLPR1 – COLPR2 (G_ST_ = 0.50) clusters, and US1A – US1B (G_ST_ = 0.54) and highest between COLPR1 – US1A (G_ST_ = 0.80), COLPR1 – US2 (G_ST_ = 0.81) and US1A – US2 (G_ST_ = 0.99).

**TABLE 2:** Population differentiation using Nei’s GST pairwise genetic dissimilarity between genetic clusters identified in *Macrophomina phaseolina*.

**Genetic cluster**	**US1A**	**US1B**	**US2**	**COLPR1**
**US1B**	0.54			
**US2**	0.99	0.64		
**COLPR1**	0.80	0.69	0.81	
**COLPR2**	0.68	0.59	0.69	0.50

All other pairwise comparisons had similar intermediate levels of differentiation when compared to any other genetic cluster (G_ST_ = 0.63–0.69). The high values of G_ST_ in all pairwise comparisons suggest very high differentiation and little migration between genetic clusters. However, US1A – US2 G_ST_ estimation, which is notably high, was limited in power due to the low levels of gene diversity (H_exp_) within these clusters. Across the 76,981 loci, there were only 76 and 255 polymorphic loci within US1A and US2 clusters, respectively. Thus, low gene diversity (H_exp_) in US1A and US2 subpopulations likely resulted in overestimation of G_ST_ in pairwise comparisons of US1A and US2 with all other clusters.

### 2.5 *Macrophomina phaseolina* is predominantly clonal in the United States and semi-clonal to mostly-clonal in Colombia and Puerto Rico

**FIGURE 4:**
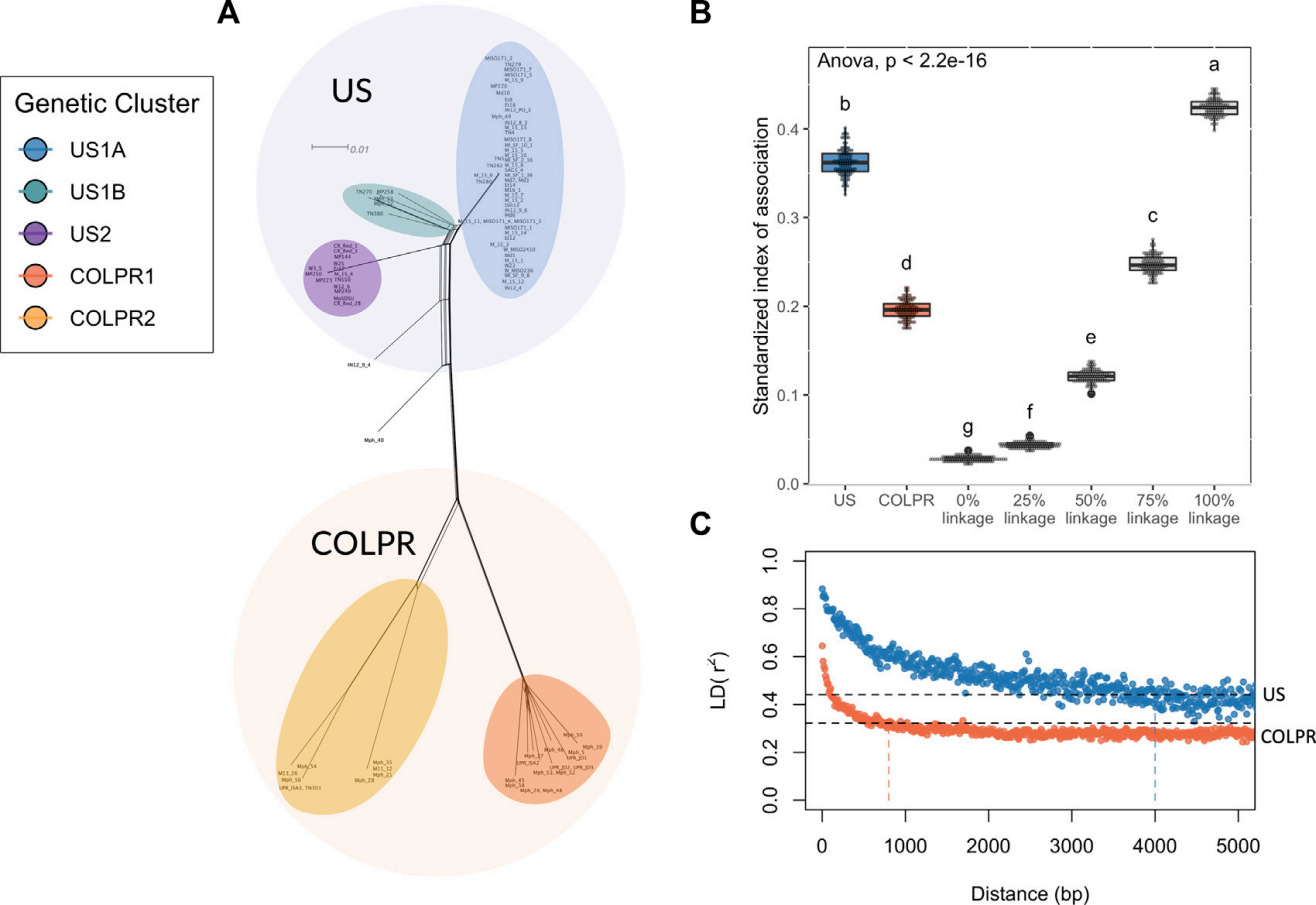
*Macrophomina phaseolina* population structure is potentially driven by clonal expansions and rapid divergence. **(A)** A reticulating phylogenetic network. Neighbor Net method was used to depict conflicting phylogenetic signal. **(B)** Estimates of linkage disequilibrium for *Macrophomina phaseolina* clades based on observed and simulated distributions of the standardized index of association (IA). Each boxplot represents the observed distribution of IA for one of the clades of *M. phaseolina*, comparedwith the distribution of IA values for simulated populations with no linkage and 25, 50, 75, and 100% linkage. The letters above each boxplot represent groupings based on Tukey’s HSD test. **(C)** Linkage disequilibrium (LD) decay for predicted populations of *M. phaseolina*, as measured by the squared correlation coefficient (r^2^) for all pairs of SNPs calculated over 50 bp windows shown for each population. The dotted black lines give the r^2^ decay to half its initial value (r^2^ = 0.44 and 0.32 in US and COLPR clades, respectively) and the vertical lines indicate the LD half-decay distance for each clade.

The predominantly star-like topology with little reticulation, in the Neighbor-Net network analysis, is consistent with a clonally reproducing population (Figure 4A). The standardized index of association (I_A_) (Brown et al., 1980) was used to estimate the degree of clonality for each of the *M. phaseolina* main populations (US and COLPR clades). The observed I_A_ distributions for each population were compared to I_A_ distributions for simulated populations with no linkage, 25%, 50%, 75% and 100% linkage. A predominantly clonal mode of reproduction was inferred in the US and COLPR populations of *M. phaseolina*. The simulated distributions and the different populations were significantly different from each other using ANOVA (F = 25,287, df = 6, *p <* 0.001). The distribution of the standardized I_A_ for the US population fell within the 75%–100% range of the linkage simulation (Figure 4B). This indicates a mostly clonal mode of reproduction with little potential for recombination. The distribution of the standardized I_A_ for the COLPR population fell within the 50%–75% range of the linkage simulation, indicating semi-clonal to mostly clonal reproduction in COLPR clades (Figure 4B). To further investigate the extent to which populations reproduce clonally, the linkage disequilibrium (LD) decay, as measured by the squared correlation coefficient (*r* ^2^) was calculated across pairs of loci for each of the clades. LD extends across a much larger distance in the US clade than in the COLPR clade, decaying over the first thousand base pairs, while in the COLPR clade LD decayed over the first hundreds of bases. LD half-decay distance, calculated as the average physical distance over which *r* ^2^ decays to half of its initial value was ∼4,000 bp for US clade and ∼800 bp for COLPR clade (Figure 4C). This indicates a high level of linkage occurs over larger regions of the genome in the US clade *versus* the COLPR clade. Importantly, although this may provide evidence for less clonal reproduction and higher recombination rates in the COLPR population, interpretation of standardized I_A_ and LD decay as associated with the frequency of recombination should be done with caution. It is possible that higher LD values did not reflect greater recombination; instead, it may be affected by lower sample size in COLPR and lower diversity in the US clade.

### 2.6 Climate contributes to SNP variation between *Macrophomina phaseolina* genetic clusters

**FIGURE 5:**
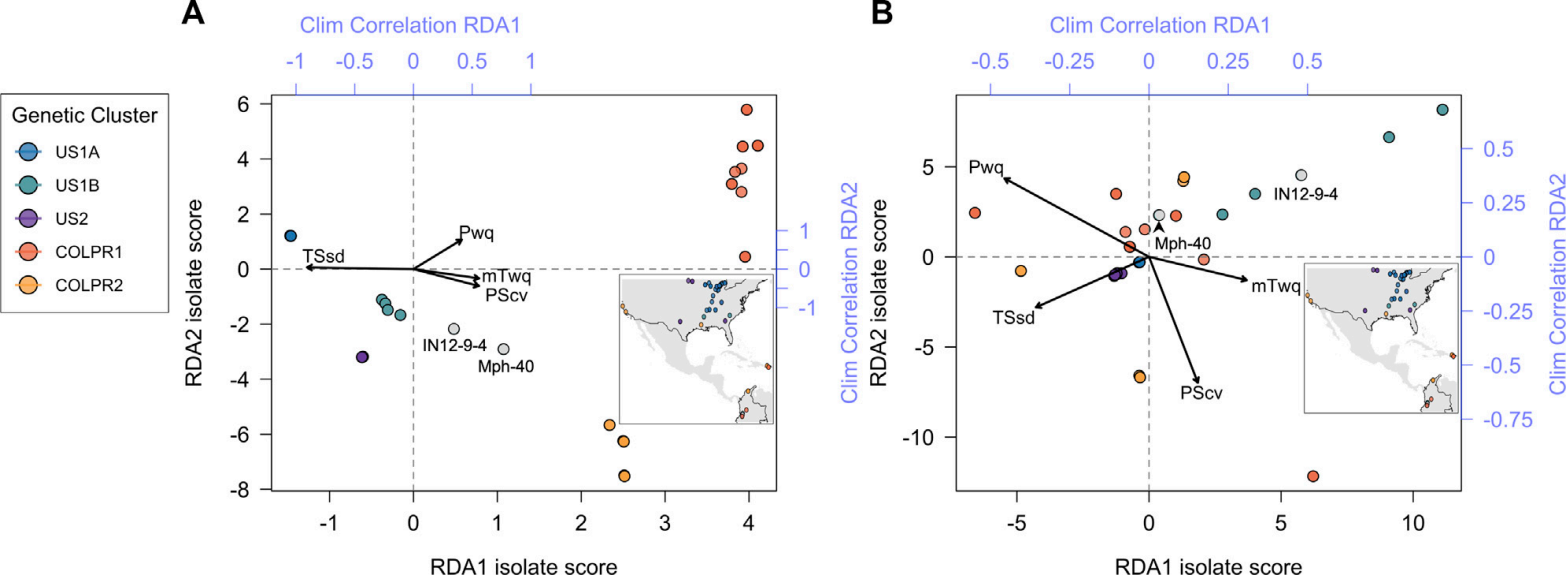
Genotype-environment association analyses support the contribution of climate variables to patterns of divergence among *Macrophomina phaseolina* populations across the United States, Colombia, and Puerto Rico. Biplot of all isolates scores for the first two RDA axes using **(A)** Simple RDA (uncondtioned) and **(B)** Partial RDA (conditioned on neutral population structure). Points are colored to show agreement with genetic clusters identified in the PCA (inset). Top and right axes (blue) indicate the correlation of each climate variable with RDA axes 1 and 2, respectively.

To test the hypothesis that climate variation contributes to genetic variation across *M. phaseolina* genetic clusters a redundancy analysis (RDA) was employed. Four climatic variables were identified as significantly predictive of genetic variation using the simple RDA model with forward variable selection. Temperature seasonality (TSsd) was the strongest predictor, explaining 28% of the variation, followed by precipitation of warmest quarter (Pwq), precipitation seasonality (PScv) and mean temperature of warmest quarter (mTwq) (Table 3). Importantly, the climatic variables included in the RDA model were selected by their presumed biological significance and to avoid collinearity with other climatic variables and thus represent a subset of the variables possibly contributing to variation based on climate. The correlation of these variables with the first two RDA axes suggests their differential contribution to SNP variation among genetic clusters (Figure 5). Spatial structure, represented as distance-based Moran’s eigenvectors maps (dbMEM), was used to identify climatic variables that are structured in space and to account for the effect of space in variance partitioning of total genomic variation. A total of three spatial variables were identified (dbMEM1-3; Supplementary Figure S5). Notably, when accounting for spatial structure (dbMEM1-3 variables), only Pwq, mTwq and precipitation of driest quarter (Pdq) were significant and accounted for 6% of SNP variation across isolates as determined with forward selection (Table 3), indicating collinearity between TSsd, PScv and space (i.e., spatially structured TSsd and PScv variation). To identify the spatial variables significantly contributing to genomic variation forward selection was used. Of the three spatial variables, only dbMEM3 significantly explained 4% of the genomic variation and described broad-scale spatial structure (Supplementary Figure S5).

**TABLE 3:** Climatic variables significantly contributing to SNP variation as determined by forward variable selection with simple RDA (redundancy analysis) and partial RDA conditioned on space.

Simple RDA (unconditioned)						Partial RDA (conditioned on space)				
Variable	*R* ^2^	Cum *R* ^2^	Cum R^2^_adj_	F-value	*p*-value	Variable	Cum R^2^_adj_	AIC	F-value	*p*-value
TSsd	0.28	0.28	0.27	36.63	0.001***	Pwq	0.03	638.88	4.96	0.002**
Pwq	0.05	0.33	0.31	6.28	0.001***	mTwq	0.04	637.67	3.06	0.004**
PScv	0.03	0.36	0.34	4.42	0.001***	Pdq	0.06	636.50	2.98	0.010**
mTwq	0.02	0.38	0.36	3.52	0.005**					

***p ≤ 0.001.

**p ≤ 0.01.

AIC = Akaike information criterion

Partial redundancy analysis (pRDA) was used to estimate the partial contribution of each set of explanatory variables (e.g., climate) while removing the effect of the remaining variable sets (e.g., neutral population structure and space). Variance partitioning with pRDA, using the LD-filtered set of 11,421 SNPs, revealed that climate (TSsd, Pwq, PScv and mTwq identified by forward selection), neutral population structure (isolate PC scores for the first three axes of a PCA using intergenic SNPs) and space (dbMEM3 variable identified by forward selection) together significantly explained 72% of the total SNP variance. Nearly half of this variance was uniquely attributable to neutral genetic structure (32%), climate (4%), or space (1%), while the other half of the SNP variation was explained jointly between the three sets of variables (Table 4). The effect of climate alone was highly significant and explained 4% of the total genetic variance after removing the effects of neutral population structure and space (Table 4). These results support the hypothesis that climate significantly contributes to genetic variation and importantly, suggests that migration, drift, and potentially additional demographic and spatially structured processes (e.g., isolation by distance), represented by neutral population structure, play a major role in shaping genomic variation in *M. phaseolina*. Moreover, the large fraction of variation common to climate, population structure and space, emphasizes the importance of accounting for confounded effects in genotype-environment associations, particularly when inferring causal associations.

**TABLE 4:** Contribution of climate, neutral population structure and space to SNP variation (11,421 unlinked SNPs) as determined by variance partitioning with partial RDA (redundancy analysis).

Partial RDA model	Inertia (variance)	*R* ^2^	*p*-value	Proportion of explainable variance	Proportion of total variance
Full model: G ∼ clim. + sp. + struct	863.8	0.725	0.001***	1.00	0.72
Pure climate: G ∼ clim. | (sp. + struct.)	46.2	0.039	0.001***	0.05	0.04
Pure structure: G ∼ struct. | (clim. + sp.)	387.0	0.325	0.001***	0.45	0.32
Pure space: G ∼ sp. | (clim. + struct.)	6.3	0.005	0.001***	0.01	0.01
Confounded climate/structure/space	424.4			0.49	0.36
Total unexplained	327.9				0.28
Total inertia	1,191.7				1.00

***p ≤ 0.001.

Note: Climate variables are temperature seasonality (TSsd), precipitation of warmest quarter (Pwq), precipitation seasonality (PScv) and mean temperature of warmest quarter (mTwq) as identified with forward selection.

### 2.7 Genotype-environment associations identify candidate SNPs for climatic adaptation

To identify loci that are potentially involved in local adaptation to climatic conditions, SNPs strongly associated with climatic variables were identified using RDA with and without accounting for population structure. Neutral population structure was used as it uniquely contributed the most to genetic variation. The RDA models, whether accounting for population structure (partial RDA) or not (simple RDA), were globally significant (*p* < 0.001) and the first three RDA axes explained most of the genomic variation associated with climate.

**FIGURE 6:**
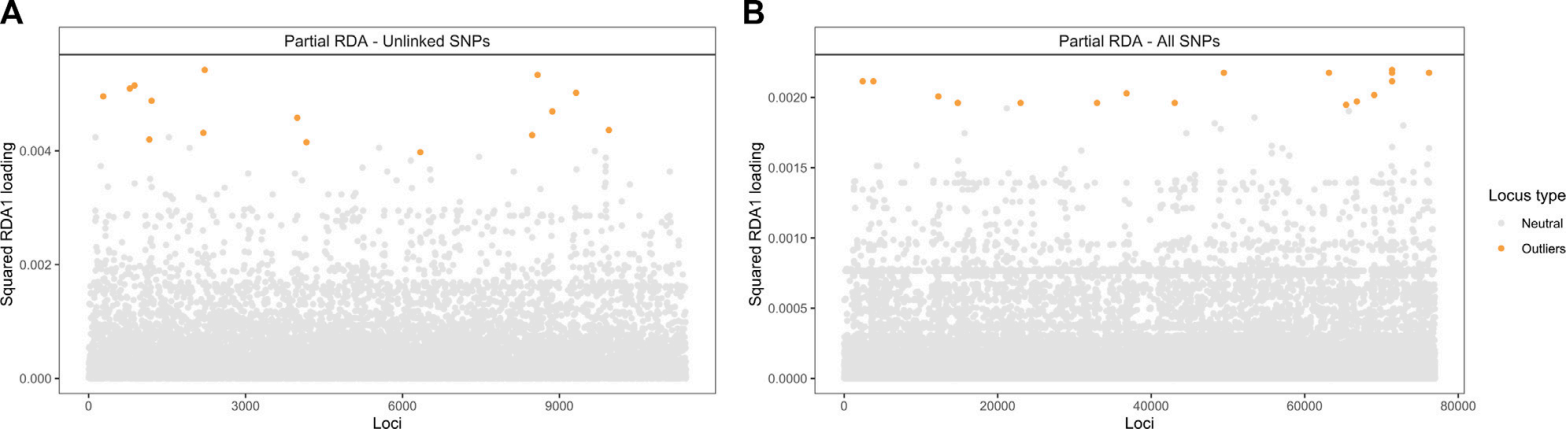
Manhattan plot of partial RDA scores. Values of squared SNP loadings for the first RDA axis conditioning on neutral population structure. **(A)** Fifteen outlier SNPs identified using 11,421 unlinked SNPs and ±3 SD from the mean and **(B)** Twenty-five using all 76,981 SNPs and ±4 SD from the mean.

The candidate adaptive loci were identified based on extreme SNPs loadings, ±3 or ±4 SD from the mean, on each of the first three axes (Forester et al., 2018). In the partial RDA models, in which the effects of population structure were removed, 49 unlinked SNPs (when using the LD-filtered set and ±3 SD from the mean; Supplementary Figure S4) and 75 SNPs (using all SNPs and ±4 SD from the mean; Supplementary Figure S5) strongly associated with climatic variables were identified along the first three RDA axes. Of these SNPs, 15 and 25 (outliers in Figure 6) were identified in the first RDA axis when using the LD-filtered set or all SNPs, respectively, and 20 (19%) in both partial models. The strongest associations include SNPs with predicted effects in the membrane-associated *753,275*-ankyrin, the *681,752*-Ksh1 and the *241,776*-protoporphyrinogen oxidase proteins. Structural modeling of the *753,275*-ankyrin protein revealed that 598 residues (96% of the sequence) was modelled with 100% homology confidence to the transient receptor potential (TRP) NOMPC (No mechanoreceptor potential C) mechanotransduction channel protein in *Drosophila melanogaster* (chain C, highest scoring template; PDB ID: 5VKQ; Extended Data Supplementary File 1). Other SNPs with top associations are located within or in physical proximity to genes related to transmembrane transport, glycoside hydrolase activity, DNA binding and the gene encoding the *28,417*-heat shock protein (Table 5; Supplementary Table S6).

**TABLE 5:** Candidate SNPs and gene models along the first RDA axis, after accounting for neutral population structure using the LD-filtered set of 11,421 SNPs.

SNP position	RDA1 loading	Climate variable	Correlation	SNP category	Distance from locus (bp)	Mycocosm gene location	Mycocosm protein ID	InterPro/KOG Desc	KOG Class/Putative function
scaffold_13:111451	-0.068	TSsd	0.90	Intergenic	3150	scaffold_13:108068-110230	753275	Ankyrin repeat	Cell wall/membrane/envelope biogenesis
scaffold_44:156076	-0.068	TSsd	0.89	Intergenic	3974	scaffold_44:152589-153590	681752	Ksh1(Protein kish)	Involved in the early part of the secretory pathway
scaffold_3:1825169	0.070	TSsd	0.62	Missense	0	scaffold_3:1824304-1825956	241776	Protoporphyrinogen oxidase	Coenzyme transport and metabolism/Heme biosynthesis
scaffold_3:1762959	-0.065	TSsd	0.62	Intergenic	1106	scaffold_3:1763965-1765572	763945	None	Unknown
scaffold_6:1672095	-0.074	TSsd	0.61	Intergenic	755	scaffold_6:1672555-1674150	726065	None	Unknown
scaffold_13:484791	-0.064	TSsd	0.59	Intergenic	563	scaffold_13:485238-487511	88264	Transcription factor domain, fungi	DNA binding/Zinc ion binding (Zn(II)_2_Cys_6_ transcription factor-related)
scaffold_1:2854576	-0.070	TSsd	0.56	Synonymous	0	scaffold_1:2854139-2855202	36408	GXWXG domain	Unknown/Putative transcription factor *Cmr1* homolog
scaffold_59:25310	-0.066	TSsd	0.51	Synonymous	0	scaffold_59:24613-27244	365205	AMP-dependent synthetase/ligase	Lipid transport and metabolism
scaffold_22:420202	-0.063	TSsd	0.50	Intergenic	4892	scaffold_22:414577-415638	787628	DUF1772 family	Unknown
scaffold_48:217394	-0.071	TSsd	0.45	Intergenic	4970	scaffold_48:211321-213431	633816	Glycoside hydrolase, family 1	Carbohydrate transport and metabolism
scaffold_3:91936	0.072	TSsd	0.39	Intergenic	1176	scaffold_3:93112-93948	735628	Allergen V5/Tpx-1-related, conserved site	Unknown
scaffold_6:1470614	0.066	TSsd	0.37	Intergenic	14457	scaffold_6:1454967-1456373	382155	Ribonuclease T2-like	RNA processing and modification
scaffold_2:1936912	0.071	TSsd	0.35	Intergenic	443	scaffold_2:1936361-1937105	643334	Thioesterase superfamily	Unknown
scaffold_42:203393	0.073	mTwq	0.29	Synonymous	0	scaffold_42:202488-204519	608404	Cytochrome P450, E-class, group I	Lipid transport and metabolism
scaffold_41:309748	0.065	mTwq	0.29	Intergenic	4841	scaffold_41:303593-305479	582790	Flavin-containing monooxygenase	Secondary metabolites biosynthesis, transport and catabolism

Because population structure could not be fully disentangled from climate (and space), as revealed in variance partitioning, the candidate loci obtained with population structure correction represent a conservative set subjected to a reduction in the detection of SNPs truly associated with climate. In the simple RDA model, without correcting for population structure, 91 candidate unlinked SNPs were identified (Supplementary Table S7). Only two SNPs were identified by both partial RDA and simple RDA models using unlinked SNPs (Supplementary Figure S6). This is in line with the high level of collinearity observed between genetic, space and climate (Table 4), and highlights the importance of accounting for confounded effects when identifying candidate loci under selection with genotype-environment associations.

## 3 Discussion

In this study, we describe the population structure of *M. phaseolina* in the continental United States, Puerto Rico and Colombia collected from soybean and dry bean fields and the contributions of climatic factors to patterns of genomic diversity among populations. We found that five distinct genetic clusters of *M. phaseolina* evolved across the United States, Colombia and Puerto Rico and evidence suggests migration between genetic clusters and countries. To date, population genetic studies in *M. phaseolina* have performed their analyses at the resolution of microsatellites molecular markers and have provided important information on genetic diversity, host and geographic associations in the United States (Baird et al., 2010; Arias et al., 2011; Koike et al., 2016). However, no population-level genomic studies have been conducted to investigate population structure in this widespread pathogen. To our knowledge, we present the first population genomics study to investigate population dynamics and the role of climate in shaping patterns of genomic variation in *M. phaseolina* at a continental and regional scale. This study uses population genomics data to identify multiple strongly differentiated genetic lineages in the United States and demonstrated novel population structure in Colombia and Puerto Rico, which previously remained unstudied. Furthermore, our results highlight the importance of within-species genetic variation in understanding pathogens adaptive response to a changing climate and offers new insight with respect to the functional roles of genomic regions potentially underlying adaptation to climate. Notably, this research provides a practical framework for genotype–environment associations studies in *M. phaseolina* and other plant pathogens with complex evolutionary and demographic histories.

The influence of the low number of loci on limiting inferences about *M. phaseolina* population structure is emphasized by recent studies that used microsatellites markers (Baird et al., 2010; Arias et al., 2011). These studies identified genetic groups in the United States; however, the genetic groups did not represent lineages (i.e., genetic groups and supported phylogenetic clades). Using population genomics, we provided strong evidence for five distinct genetic clusters of *M. phaseolina* and revealed that genomic variation in this globally distributed pathogen was consistent with a population hierarchically structured at a broad subcontinental regional extent. Two genetically differentiated *M. phaseolina* clades at the United States and Colombian-Puerto Rican geographical level and five distinct genetic clusters representing finer population structure within each of these clades were identified. These genetic clusters, except for US1B, represent strongly supported phylogenetic clades and likely represent different evolutionary lineages of *M. phaseolina*. This distinction is important because the identification of lineages allows for the inference of ecological and evolutionary processes in a population-specific manner and underscores the potential for local adaptation in *M. phaseolina* populations.

Our results provide support for regional clustering within the United States and a lack of strong grouping at a state level, also observed in previous studies based on microsatellite data (Baird et al., 2010; Arias et al., 2011). The US1A cluster, found in the East North Central and Central region, expands previous studies confirming that isolates collected from soybean in these regions represent a largely homogeneous population (Arias et al., 2011). This is supported by low gene diversity and pairwise genetic distances found in the US1A genetic cluster in agreement with low diversity detected with microsatellite markers in soybean isolates collected mostly in Tennessee and Missouri (Arias et al., 2011) and midwestern states (group III; Baird et al., 2010). The US2 genetic cluster found in Northern Great Plains and South United States regions grouping isolates from Minnesota, South Dakota, Texas, and Georgia is partially consistent with Baird et al. study. Isolates from these states along with isolates from North Dakota represent the majority of a subcluster of group I in Baird et al. Like in the US clusters, grouping at broad geographic regions was observed in COLPR1 and COLPR2 clusters. Both COLPR1 and COLPR2 clusters grouped isolates from locations across Colombia and Puerto Rico. In COLPR2, isolates from California and Louisiana grouped closely to isolates from Colombia and Puerto Rico. Although the small sample size from these states (only two isolates collected from strawberry in California and one isolate from soybean in Louisiana) demands that this grouping be reassessed once more isolates are included from these states and hosts in future studies. The clustering of isolates from widespread geographic regions observed in COLPR2, as well as in US1A and US1B clusters, suggests a role for migration in structuring *M. phaseolina* populations. These results better align our understanding of *M. phaseolina* population structure with a metapopulation model, that predicts regional persistence of populations while local populations are unstable and connected by some level of migration (Hanski, 1998; Milgroom, 2015). The metapopulation dynamics view expands the interpretation of past *M. phaseolina* population structure studies while providing a conceptual basis for the design of future studies.

The presence of multiple distinct genetic clusters in the United States and higher genetic diversity in COLPR clusters led us to inquire about whether Colombia and Puerto Rico may serve as potential source populations for US populations. In the rooted ML phylogeny, the reconstruction of COLPR clusters as outgroups to US clusters support this hypothesis. Furthermore, across all analyses we found indications that US1B may serve as a sink population for Colombia and Puerto Rico populations. The US1B genetic cluster grouped isolates from Mississippi and South Carolina along with two Colombian isolates and was the most genetically diverse of the US clusters. Further, US1B was positioned centrally in PCA space, basal to US1A cluster in the rooted ML phylogeny and was less differentiated, along with COLPR2, from all other clusters based on G_ST_ values. Finally, in DAPC analysis, US1B isolates clustered with IN12-9-4 and Mph-40 isolates, which are reconstructed intermediate between US and COLPR clades in the rooted ML phylogeny and as admixed in spatial population structure analyses. Although, the high diversity in US1B may be reflective of the grouping of comparatively few isolates from different geographic regions in this cluster. However, when all data are considered, it suggests the US1B cluster geographic region as a potential route of introduction of isolates from Colombia or Puerto Rico to the United States. More isolates from the United States and other countries would need to be included in future studies to test this hypothesis.

The discrete population structure observed between US and COLPR clades, provides compelling evidence for isolates in each clade drawing ancestry from different ancestral populations. A plausible explanation, supported by our results, for this different ancestry would be a demographic event such as a rare long-distance migration (e.g., introduction event) from the COLPR clusters, leading to a recent bottleneck in the US populations. The high probability assignments observed in US clusters may be consistent with the expected strong recent genetic drift in bottlenecked populations (Lawson et al., 2018). In this scenario, we speculate that the diversity in US clusters represent a subset of the diversity of the COLPR genotypes found in Colombia and Puerto Rico. At the finer genetic cluster population structure, isolation by distance provided a potential explanation for the continuous genetic differentiation in spatial population structure analyses. Although, isolation by distance patterns may be observed as part of a variety of underlying biological processes and demographic scenarios (Sexton et al., 2014; Milgroom, 2015), it is possible that these patterns reflect a scenario of restricted dispersal in the context of divergence following clonal expansions in the US genetic clusters. For example, both US1A and US2 genetic clusters are found in Michigan, Wisconsin, and Kentucky, supporting dispersal of isolates among these states. However, high population differentiation indicated by high G_ST_ values between genetic clusters, suggest substantial restriction to gene flow. Given the soilborne nature of *M. phaseolina* and limited natural dispersal ability but high potential for anthropogenic mediated dispersal, restricted events of dispersal associated to seed, plant material or farm equipment at limited distances relative to the geographic range of the genetic clusters, seems a likely occurrence (Baird et al., 2010). Similar isolation by distance patterns has been observed in other soilborne fungal and oomycete pathogens with restricted long-distance dispersal (Grünwald and Hoheisel, 2006; Milgroom et al., 2016).

Diversity was found to be further reduced in US1A genetic cluster as compared to all other clusters. Low diversity and high differentiation are signatures of genetic drift but also selection. If reduced diversity in the US1A genetic cluster was consistent with a clonal expansion following a bottleneck, the divergence and marked low diversity could reflect both genetic drift and selection. Genetic drift is expected to have substantial effects on pathogen populations, because migrations resulting in founder effects and reduced population sizes associated with pathogens survival in soil (Milgroom, 2015). Additionally, we speculate that climatic conditions, particularly strong fluctuations in temperature in the northern United States, could impose strong selection on *M. phaseolina* populations in this region. Overall, we believe the genomic signals of discrete and continuous structure that differentiate *M. phaseolina* populations could be reflective of a complex demographic and evolutionary history. Therefore, alternative demographic scenarios, including one of multiple independent introductions, should be considered in future studies ideally applying demographic modelling with a broad geographic and temporal distribution of isolates.

Across all analyses we found support for Colombia and Puerto Rico as potential sources for United States *M. phaseolina* populations. Genetic diversity between countries also supported this hypothesis. Whereas Colombian isolates were significantly more diverse than United States isolates, diversity in Puerto Rico was intermediate and not significantly different from United States or Colombia. These findings may be consistent with the idea of Middle or South America as putative centers of origin for *M. phaseolina* and with its introduction to North America as part of historical crop migrations. For example, common bean Middle American origin, domestication centers in Middle America and South America (Bitocchi et al., 2017) and later movement to the United States via the Caribbean, Central and Eastern United States (Kelly, 2010), makes likely an explanation for *M. phaseolina* introduction to the United States in bean seeds. Pathogen geographic origins have been associated with the centers of diversity of their major crop host. Nonetheless, pathogen origin associated with their hosts’ wild relatives, have been also observed in some plant pathogens. For example, a genetically diverse and sexually reproducing population of *Phytophthora infestans* was found in central Mexico consistent with this pathogen’s origin in a secondary center of potato (*Solanum tuberosum*) diversity and potentially involved in a host jump from native *Solanum* species (Goss et al., 2014). Given *M. phaseolina* host generalist nature, a strict host-pathogen coevolution scenario is not expected (Slippers and Wingfield, 2007), obscuring inferences about its center of origin. In Kansas, isolates collected from wild tallgrass prairie were found more diverse than isolates from maize, soybean and sorghum crops (Saleh et al., 2010). This finding may indicate *M. phaseolina* presence in the United States precedes the introduction of agriculture or it may be explained by connectivity dynamics between natural and agricultural ecosystems contributing to patterns of diversity in *M. phaseolina* populations from these ecosystems (Saleh et al., 2010). Thus, the origin and evolutionary history of *M. phaseolina* is likely more ancient and complex than could be tested with the isolates included in this study, and future studies may benefit from considering the potential involvement of host adaptation from wild hosts.

Genotype tracking provided compelling evidence for migration among the United States, Colombia, and Puerto Rico. The MLL consisting of the Colombian isolate Mph-49 and several isolates from the United States clustering in US1A, along with the high clonality found in this cluster and the significantly high diversity in Colombia, makes a Colombian source likely. Similarly, the MLL shared between Colombia and Puerto Rico and the MLL between Puerto Rico and Louisiana support migration between countries. Alternatively, the same MLLs could have been introduced independently to United States, Puerto Rico, and Colombia, potentially from an ancestral and more diverse population not included in this study. Although this scenario seems less likely, it remains a possibility. Given that besides historical crop migrations, migration as part of international seed exchange is a likely occurrence in *M. phaseolina*, as in other seedborne species and latent pathogens of the Botryosphaeriaceae family (Sakalidis et al., 2013; Crous et al., 2017), we believe that *M. phaseolina* has been spread at least intercontinentally, possibly globally, through seed. However, time, frequency, and directionality of migration between United States, Colombia, and Puerto Rico, and the potential for multiple introductions would need to be examined in future studies.

Although various population genetic studies in *M. phaseolina* have found patterns of host associations (Jana et al., 2005; Baird et al., 2010; Arias et al., 2011; Koike et al., 2016; Reznikov et al., 2018; Burkhardt et al., 2019), our results did not find that genetic variation is associated with host in the two major US clusters. Soybean and dry bean isolates grouped together in US1A and US2 clusters. Given that most previous studies support some degree of host preference, and genomic evidence for genes uniquely present in the *M. phaseolina* strawberry genotype further support host preference (Burkhardt et al., 2019), we suspect that our sampling scheme was not enough to capture clear associations to a plant host. A clear limitation in our study was that the host origin was confounded with geographic origin, except for Michigan where isolates were sampled from both soybean and dry bean. The independent grouping of host might also reflect crop rotation and equipment practices implemented in fields. Additionally, our data may reflect that the sampled hosts are both legumes. Genetic similarity has been found to be greater among isolates collected from the same host than from hosts in different families (Su et al., 2001; Saleh et al., 2010). These results do, nonetheless, have important practical implications for soybean breeding resistance to charcoal rot. In the US1A cluster, the high genetic similarity of isolates collected from soybean and dry bean, may indicate that the use of one or few isolates collected from these crops throughout East North Central and Central United States regions may suffice for resistance screening of soybean breeding material. An important limitation to this assumption is that we use a single reference genome approach to characterize genetic diversity and thus accessory genes and other structural variation potentially involved in pathogenesis are not considered (Bertazzoni et al., 2018).

Importantly, the dry bean diversity in research plots from which Colombian and Puerto Rican isolates were collected is a factor likely contributing to their higher genetic diversity as compared to United States isolates. In research plots, multiple lines are continually evaluated as part of breeding programs, in contrast to commercial fields in which a single or few varieties are used. This coupled with climatic conditions in Colombia and Puerto Rico that favor year-round inoculum presence in crop residue represent important considerations when interpreting isolate genetic diversity in relation to host origin.

The population structure results suggest that *M. phaseolina* populations lay in-between the clonality-recombination spectrum (Smith et al., 1993). Furthermore, our results suggest that this may occur in a population-specific manner. On one side of the spectrum, we found *M. phaseolina* to have a markedly clonal population structure (Milgroom, 2015). First, most of the intraspecific genetic variation in *M. phaseolina* is explained by differences between clades and genetic clusters, while low genetic variation was observed within genetic clusters. Second, the occurrence of nearly identical genotypes (i.e., MLLs) from widespread geographic locations found in *M. phaseolina* is in line with a markedly clonal population structure (Milgroom, 1996; Milgroom, 2015). On the other end of the spectrum genotypic diversity, network analyses and measures of linkage among loci provided support for recombination within some of the genetic clusters. High levels of genotypic diversity is one of the characteristics reflective of recombination in fungal populations (Milgroom, 1996). The higher genotypic diversity (eMLLs) in US1B, US2, and COLPR clusters, may be consistent with the occurrence of recombination in these clusters. Network analyses account for recombination by allowing to infer homoplasy caused by recombination. The boxes between isolates within genetic clusters in the network and the PHI test supporting recombination within all clusters except for US1A, strengthen this hypothesis. The index of association, I_A_, revealed an overall high degree of linkage among SNP markers, in line with a pathogen that reproduces clonally. However, the observed I_A_ values in the COLPR clade and LD decaying faster in COLPR than in US populations, support the potential occurrence of recombination among isolates within COLPR clusters. Although the problem of smaller sample size in COLPR clusters should be at least partially accounted for by using simulations in IA analysis and clone-corrected data in LD-decay analysis, particularly half-decay LD values should be interpreted with caution and examined in future studies to determine the extent of recombination in *M. phaseolin*a populations.

These results are consistent with the population structure model that lays in between the “strictly clonal” and “epidemic” structure proposed by Smith et al. (1993), in which frequent recombination does not occur between isolates in separate branches of an evolutionary tree but it occurs between isolates within a given branch (Smith et al., 1993). These models have been used to describe the population structure of plant pathogens with mixed modes of reproduction or inferred recombination (Grünwald and Hoheisel, 2006; Milgroom et al., 2014; 2016; Milgroom, 2015). While little is known about the occurrence of recombination in *M. phaseolina*, recent studies have started to shed light on potential recombination mechanisms involving parasexuality (Pereira et al., 2018) and horizontal gene transfer mediated by giant mobile genetic elements (Gluck-Thaler et al., 2022). Whether other potential recombination mechanisms occur, and the frequency of recombination in *M. phaseolina* remains an important and exciting area of study.

Partial RDA revealed that nearly half of the SNP variance is confounded between neutral genetic structure, climate, and space. This means that this fraction of the variance cannot be statistically associated to a direct effect of any single set of variables. Importantly, the effects of population structure and space often cannot be independently disentangled from spatially structured process (e.g., IBD) or spatially structured environmental variables (Lasky et al., 2015). This study, while highlighting the challenges in assessing genotype-environmental associations, provided an assessment of the fraction of confounded variance and allowed us to start disentangling the effects of climate, spatial, and population structure on genomic variation in *M. phaseolina* populations. The genotype-environment association analyses using partial RDA support our hypothesis that local climatic differences contribute to patterns of adaptive divergence among *M. phaseolina* populations across the United States, Colombia, and Puerto Rico. Seasonal variation in temperature and precipitation of warmest quarter, were the primary climatic variables associated with variation of candidate adaptive loci without and after accounting for neutral genetic population structure, respectively. We found SNPs within or in physical proximity to genes with functional annotations related to transmembrane transport, glycoside hydrolase activity and DNA binding. In fungi, genes involved in these activities are known to be important in responses to environmental stressors including temperature, water availability, and oxidative stress (Aguilera et al., 2007; Gasch, 2007; Branco et al., 2016). Similarly, among the candidates, we found the *241,776*-protoporphyrinogen oxidase protein, involved in heme biosynthesis and the putative small heat shock protein *28,417*-*Hsp20*. Heme has been shown to regulate several mechanisms during cold shock in *Saccharomyces cerevisiae* (Abramova et al., 2001) while *Hsp20* proteins have been found involved in fungal thermal stress response to both heat and cold (Wu et al., 2016; Wang et al., 2021).

The SNP with the highest correlation with temperature seasonality was located upstream to the *753,275*-ankyrin repeat protein (Table 5). We found that *M. phaseolina 753,275*-ankyrin protein is homologous to the TRP NOMPC mechanotransduction channel in *D. melanogaster* (Jin et al., 2017). Ankyrin family proteins link membrane proteins, including ion channels, to microtubules of the cytoskeleton by binding of its ankyrin repeat domain. The ankyrin proteins in the NOMPC channel links a displacement of the cytoskeleton to the channel opening, translating external stimuli into intracellular signals (Jin et al., 2017). Moreover, the TRP1 (transient receptor potential 1) ion channel from the alga *Chlamydomonas reinhardtii*, which shares structural homology to the TRP NOMPC channel, was found to act as thermal sensor, with ankyrin proteins mediating the channel opening in response to increased temperature (McGoldrick et al., 2019). Although there is no structural or functional characterization of the *M. phaseolina 753,275*-ankyrin protein, it represents a promising candidate to investigate a potential temperature-related mechanism for environmental stimuli transduction. These findings are consistent with the established roles of proteins in environmental stress responses both specific to fungi and conserved across the tree of life. Although our results cannot confirm whether SNPs are the causal mechanism, the candidate genes could be used in future functional studies. Additionally, common garden experiments could provide support for local adaptation to climate in *M. phaseolina*.

Overall, our observations point to a scenario in which *M. phaseolina*, as other plant pathogens with clonal population structures, is structured in a subcontinental regional stable manner in the face of instability at local scales in line with the metapopulation dynamics perspective. These results are consistent with a scenario of evolution after migrations driven by divergence following clonal expansions. The presence of MLLs across countries underscores the potential for a large influence of anthropogenic migration introducing *M. phaseolina* to new environments. The association of genetic divergence with climatic variables and putatively adaptive functions of the genes with SNPs strongly associated that would hypothetically benefit *M. phaseolina* in specific environments, is consistent with potential selection imposed by specific climatic variables. Future studies will be needed to identify the degree to which distinct genetic groups reflect their adaptation to host and climate. Such analyses will benefit from a global sampling collected from diverse hosts in conjunction with multiple reference genomes sequenced with long-read technologies that will allow further characterization of the role of genomic variation, including structural variation, in *M. phaseolina* adaptation to host and the climatic environment.

This knowledge expands the impact that spatial population genomics and genotype-environment associations can have on our ability to characterize adaptive potential in plant pathogens by identifying candidate genes and presents a preliminary and complementary approach to the forward-genetics and phenotypic characterization approaches. The ability to identify candidate genes at a population specific level in a clonal pathogen presents an opportunity to evaluate candidate genes in a population specific manner, which represents a powerful approach specially in clonal pathogens in which unusually high levels of linkage prevent the application of genome scan methods. Additionally, the RDA approach could be applied using candidate adaptive genetic markers to predict pathogens’ “adaptive landscape” representing its adaptive variation for any environment across a geographic range (Capblancq and Forester, 2021). As climate and agricultural challenges become more demanding, the characterization of pathogen adaptation capabilities enabled by population genomics should become increasingly utilized for plant disease risk prediction models specially under adverse future climate scenarios. In conclusion, our study emphasizes the importance of population genomics for identifying distinct genetic groups and uncovering potential recombination and population-specific adaptation related to climate in fungal plant pathogens, specifically in *M. phaseolina*. The spread of these adaptations among populations through gene flow at local scales or transcontinental introductions could create challenges in managing charcoal rot, as selection pressure could lead to the emergence of highly adapted and virulent pathogen populations. Hence, it is crucial to monitor the emergence and introduction of pathogens with novel adaptations. Our findings provide a foundation for future research with practical applications for management of charcoal rot, such as developing plant disease risk prediction models, informing the development of resistant crop varieties, and regulating the global movement of *M. phaseolina*.

## 4 Methods

### 4.1 Isolate collection and DNA preparation

A total of 95 *M. phaseolina* isolates were obtained from culture collections, as well as roots or lower stems of soybean and dry bean plants in production fields (Supplementary Table S1). Isolates were originally collected in the United States, 70; Colombia, 20; and Puerto Rico, 5 and selected to reflect a latitudinal range and climatic variation (Figure 1). There were 52 isolates collected from soybean across a latitudinal range in 13 US states, including 38 isolates from a previous study (Sexton et al., 2016). Forty isolates were collected from dry bean grown in Michigan, Puerto Rico and Colombia. Isolates from Michigan were collected from 2011 to 2017 as part of root rot surveys conducted in soybean and dry bean (Rojas et al., 2016; Jacobs et al., 2019). Isolates from Puerto Rico and Colombia were collected from research plots at the University of Puerto Rico (UPR) and at the International Center for Tropical Agriculture (CIAT) and are currently maintained at the UPR and CIAT Plant Pathology Laboratory culture collections. Two strawberry isolates collected from California and one isolate from Ethiopian mustard (*Brassica carinata*) were included as host outgroups. Cultures were routinely grown on potato dextrose agar (PDA; Acumedia, Lansing, MI) medium.

For genomic DNA extraction, four 5-mm plugs taken from the edge of a single hyphal tip culture were used to inoculate 50 mL of potato dextrose broth amended with chloramphenicol (50 mg/L). The broth was incubated for 7–9 days at room temperature. Mycelia were harvested, lyophilized for 24 h and ground using a FastPrep FP120 homogenizer (BIO 101 Savant Instruments, Hobrook, NY). Genomic DNA was extracted from the lyophilized tissue using a modified SDS-based method; briefly, 50 mg of ground mycelia were mixed in lysis buffer (3% SDS (w/v); 100 mM Tris-HCl, pH 8.0; 50 mM EDTA, pH 8.0) followed by phenol/chloroform DNA extraction. The identity of all isolates was confirmed by multigene DNA analysis of the Internal Transcribed Spacer regions for the nuclear rDNA operon (ITS), part of the Translation Elongation Factor (TEF-1α) gene region, and part of the actin (ACT) gene region according to (Sarr et al., 2014). Maximum likelihood analysis of the combined sequence alignment placed all the isolates tested in the *M. phaseolina* cluster. A full heuristic search using the first ten most parsimonious trees and the Neighbor-joining tree as starting trees with 100 random sequence additions was performed in PAUP v4.0b10 (Swofford, 2003), to find the maximum likelihood tree (Supplementary Figure S7).

### 4.2 Whole genome sequencing and variant calling

Genomic libraries were constructed and each of the isolates were whole-genome sequenced to 23X coverage using a 150 base-pair paired-end strategy on the Illumina HiSeq 4,000 platform at the Michigan State University Research Technology Support Facility Genomics Core (East Lansing, MI). The libraries were prepared using the Illumina TruSeq Nano DNA Library Preparation Kit HT. The resulting sequences were quality assessed using FastQC (Andrews, 2010) and cleaned using Cutadapt v1.16 (Martin, 2011), with the following parameters: f fastq, -q 20,20, --trim-n, -m 30, -n 3, -a AGA​TCG​GAA​GAG​CAC​ACG​TCT​GAA​CTC​CAG​TCA​C, -A AGA​TCG​GAA​GAG​CGT​CGT​GTA​GGG​AAA​GAG​TGT​AGA​TCT​CGG​TGG​TCG​CCG​TAT​CAT​T. After initial quality filtering, the remaining sequences were aligned to the *M. phaseolina* reference genome Macpha1 (JGI Mycocosm, MPI-SDFR-AT-0080 v1.0) using bwa-mem (Li, 2013). The isolate used for the Macpha1 reference genome was collected from natural *A. thaliana* populations in France (Mesny et al., 2021). The mapping statistics, genome alignment rate and genome coverage were assessed with SAMtools flagstat (Li et al., 2009). Alignments were sorted and indexed using SAMtools (Li et al., 2009). After mapping, duplicate reads were identified using MarkDuplicates and removed during the variant calling step.

Single nucleotide polymorphisms (SNPs) of all 95 isolates were predicted using the Genome Analysis Toolkit (GATK) v4.0 (McKenna et al., 2010). Initially, SNPs were called individually with GATK’s HaplotypeCaller. GVCF files were combined, and common SNPs jointly identified using CombineGVCFs and GenotypeGVCFs programs. The later using the -new-qual parameter. The combined vcf file was quality filtered using vcfR v1.10.0 package (Knaus and Grünwald, 2017) in R v4.0.0 (R Core Team, 2019). To be included in the high-quality set, SNPs were filtered to remove SNPs with a minimum read depth (DP) of <4x and greater that the 95th percentile of each sample DP distribution and exclude SNPs with minimum threshold mapping quality (MQ < 60) and minimum allele frequency (MAF <0.02) which corresponds to the allele presence in at least two isolates. Only variants with no missing data were retained, which corresponds to positions with 0 missing data for all the sequenced isolates. The final high-quality dataset was used in all subsequent analysis. The final vcf was annotated using SnpEff v5.0c (Cingolani et al., 2012b) and a vcf containing only SNPs in intergenic regions was created using SnpSift v5.0c (Cingolani et al., 2012a).

### 4.3 Phylogenomics and population genetic structure

The population structure was inferred according to the results from both model-based and model-free clustering methods and phylogenetic inference. The phylogenetic tree was inferred from the full set of high-quality SNPs among the 95 *M. phaseolina* isolates in RAxML-NG v1.01 (Kozlov et al., 2019). The RAxML analysis was performed using the “-all” option which conducted 20 maximum likelihood inferences on the original SNP alignment, standard bootstrapping with automatic determination of the number of replicates (Felsenstein’s bootstrap, FBP; MRE-based bootstopping test) and the subsequent maximum likelihood search. The General-Time-Reversible (GTR) model of nucleotide substitution with GAMMA model of rate heterogeneity and correction for ascertainment bias (GTR + G + ASC_LEWIS) was used. The best-scoring ML tree was used for optimizing all model and branch length parameters and model evaluation. A model-free dimensionality-reduction approach, principal component analysis (PCA), and discriminatory analysis of principal components (DAPC) were also conducted on the full set of SNPs using adegenet package (Jombart, 2008; Jombart and Ahmed, 2011) in R 4.0.0 (R Core Team, 2019). To infer population dynamics and reconstruct a rooted *M. phaseolina* phylogeny, the Macpha1 (JGI Mycocosm, MPI-SDFR-AT-0080 v1.0) reference genome was used as outgroup taxon. Maximum likelihood analysis was run in RAxML-NG v1.01 using the “-all” option with automatic bootstrap replicates and the GTR + G + ASC_LEWIS substitution model.

### 4.4 Spatial genetic structure

Bayesian clustering of allele frequencies was implemented in conStruct (Bradburd et al., 2018). To assess whether population structure was well described by modelling isolates as admixtures between multiple discrete genetic groups or by both discrete and continuous genetic structure, spatial analysis of population structure was conducted using conStruct (Bradburd et al., 2018). Spatial analysis in conStruct, accounts for isolation by distance by allowing genetic differentiation to increase with geographic distance within discrete genetic groups (layers, K). The data was analyzed treating individual isolates as the unit of analysis, using the spatial models setting K between 1 and 7 with 20,000 iterations, and compared these models using cross-validation with 10 replicates. For cross-validation, 90% of loci were used to fit the model and the remaining loci for model evaluation. A geographically constrained least-squares method as implemented in TESS3 (Caye et al., 2016), was used to estimate ancestry coefficients and create interpolation maps based on the coefficients. TESS3 uses a spatially explicit algorithm that can be considered model-free. The algorithm was run using the function “tess3” with K between 1 and 7 and 10 replicates.

### 4.5 Population genetic and genotypic diversity

For each clade and genetic cluster, gene diversity (Nei, 1978) was calculated using the Hs function in the adegenet package (Jombart, 2008; Jombart and Ahmed, 2011). The median estimates of pairwise genetic distance and genotypic diversity indices were calculated within each clade and genetic cluster using the R package poppr v2.9.0 (Kamvar et al., 2014). Genotypic diversity was assessed by calculating the number of multilocus genotypes (MLGs). A MLG was defined as a unique combination of the 76,981 SNPs. MLGs were collapsed into larger groups called multilocus lineages using the average neighbor algorithm and a Prevosti’s distance threshold of 0.0001 (bitwise.dist function; Kamvar et al., 2015). Rarefaction was used to correct for uneven sample sizes using the R package vegan v2.5–6 (Oksanen et al., 2019) and obtain the number of expected MLGs and MLLs (eMLG and eMLL) at the lowest common sample size (i.e. 25 for clades and 5 for genetic clusters). Genotypic diversity indices, Shannon-Wiener Index (H*), Stoddart and Taylor’s Index (G*), Simpson’s index (lambda*) and evenness (E5*) (Grünwald et al., 2003), were calculated using the R package poppr v2.9.0 (diversity_ci function; Kamvar et al., 2014) based on the number of MLLs in each clade and genetic cluster and correcting for unequal sample sizes based on rarefaction. The function mlg. crosspop in poppr was used to detect the presence of MLGs occurring across populations. Migration was inferred by tracking MLGs across genetic clusters, referred here as genotype flow (McDonald and Linde, 2002).

### 4.6 Population differentiation between genetic clusters and countries

The F_ST_ analog, G_ST_ (Nei, 1973) was calculated from clone-corrected data using vcfR (Knaus and Grünwald, 2017) to infer differentiation among genetic clusters. To describe the population dynamics between the United States, Puerto Rico and Colombia, the degree of genetic differentiation across *M. phaseolina* samples was measured hierarchically by genetic clusters within clades. Analysis of molecular variance (AMOVA) based on the quasi-Euclidean distance matrix was conducted in poppr v2.9.0 (Kamvar et al., 2014). AMOVA estimates the number of differences summed over loci based on a matrix of distances between individuals and covariance components are used to calculate Φ fixation indices for each hierarchical level, among clades, among genetic clusters and within genetic clusters. Significant differences of fixation indices were determined by 1,000 random permutations (Grünwald and Hoheisel, 2006).

### 4.7 Recombination and clonality

To account for potential intraspecific recombination among *M. phaseolina* isolates, a phylogenetic network was built using the Neighbor-Net algorithm as implemented in SplitsTree4 v4.16.1. The extent of clonality was tested by calculating the proportion of significant linkage between pairs of loci, by computing the standardized index of association (I_A_, Brown et al., 1980) for each of the main populations (US and COLPR) using poppr v2.9.0 (Kamvar et al., 2014). Linkage disequilibrium is expected in asexual or inbreeding populations and I_A_ values close to zero are expected for outcrossing populations (Burt et al., 1996). The observed I_A_ distributions for each population were compared to five simulated recombined distributions (0%, 25%, 50%, 75% and 100% linkage) generated among 76, 981 loci and 48 samples (corresponding to the median population size of the two clusters). The observed and simulated I_A_ values were tested for normality using the Shapiro-Wilk’s normality test and an analysis of variance (ANOVA) was conducted to test for significant differences among the distributions. Pairwise comparisons between the I_A_ simulated distributions and for each population were tested for difference with Tukey’s HSD test in R. The extent of clonality was correlated to clonal (100%), mostly clonal (75%), semiclonal (50%, 25%) or sexual (0%) modes of reproduction. Linkage disequilibrium (LD) decay rate was estimated using the physical distance over which LD decays to half its initial value, as measured by the squared correlation coefficient (*r* ^2^). The linkage disequilibrium decay was calculated for each clade using the correlation coefficient (*r* ^2^) in TASSEL v5 (Bradbury et al., 2007) within a window of 50 sites among SNPs using the clone-corrected dataset (79 MLGs). The mean *r* ^2^ values, representing the correlation between alleles at two loci within 10 bp of physical distance, were then plotted in R 4.0.0 (R Core Team, 2019).

### 4.8 Climatic data

For each isolate, the 19 standard bioclimatic variables available at the WorldClim2 database (Fick and Hijmans, 2017) were obtained using ‘getData’ function from raster R package (Hijmans, 2022). All variables are the average for the years 1970–2000 and were obtained at a spatial resolution of 2.5 min (∼21.5 km^2^). We used data at a resolution of 2.5 min (∼21.5 km^2^), because it corresponds with our sampling design (single isolate samples rather than populations) being at a field or county scale. Coarser resolutions could combine multiple sampling locations into a single spatial grid and finer resolutions (30-s or <30-s), while this may be important for structuring patterns of genetic variation within populations, these data are less suitable for our sampling design and focus on regional to continental-wide patterns. We reduced the number of climatic variables from 19 to five to account for collinearity among them (|r| > 0.7) and to represent our hypothesis about the most important factors potentially driving selection. Diseases caused by *M. phaseolina* are more prevalent during hot and dry conditions, therefore temperature and precipitation variables were included. The selected climatic variables were: BIO18 = Precipitation of Warmest Quarter, BIO15 = Precipitation Seasonality (Coefficient of Variation), BIO17 = Precipitation of Driest Quarter, BIO10 = Mean Temperature of Warmest Quarter and BIO4 = Temperature Seasonality (standard deviation *100). Each bioclimatic variable was scaled, centered, and evaluated for inclusion using forward selection with 10,000 permutations using adespatial R package (Dray et al., 2022).

To account for underlying spatial structure (autocorrelation) and reduce spurious GEA, distance-based Moran’s eigenvector maps (dbMEM) were generated using sample coordinates in the quickMEM R function (Borcard et al., 2018). The dbMEMs are a matrix of axes that capture spatial patterns from multiple angles rather than just a latitudinal or longitudinal vector. Only significant dbMEM axes were selected using forward selection with 1,000 permutations. A simple RDA model and partial RDA model conditioning on space, using only significant dbMEMs, were used to identify the climatic variables significantly contributing to genomic variation and those structured in space.

### 4.9 Variance partitioning and outlier loci identification

To identify potentially adaptive loci, associations between genetic data (loci) and climatic variables hypothesized to drive selection were evaluated using a multivariate method, redundancy analysis (RDA, as implemented by (Capblancq and Forester, 2021). RDA simultaneously tests multiple loci that covary in response to climatic variables. Partial RDA models were used for variance partitioning and outlier loci identification while correcting for neutral genetic population structure. Variance partitioning analysis was performed with linkage-disequilibrium (LD)-filtered (*r* ^2^ > 0.9) dataset of 11,421 SNPs. The independent contribution of each set of explanatory variables: climate, neutral population structure or space, was assessed while removing the effect of the remaining variable sets using partial RDA. In outlier loci identification, using a partial RDA is recommended to reduce the number of false-positive detections particularly in scenarios of multilocus adaptation when selective agents are unknown (Forester et al., 2018). On the other hand, partial RDA can lead to high false-negative detections when variance is confounded between climatic variables and neutral population structure (Capblancq and Forester, 2021). Candidate adaptive loci were identified using simple and partial RDA models to examine the extent of this issue. A partial RDA model conditioning on neutral population genetic structure was used for candidate outlier SNPs detection. Outlier loci were identified in the three significant constrained axes as the SNPs having loadings ±3 or ±4 SD from the mean score of each constrained axis using both the LD-filtered set of 11,421 SNPs and the full set of 76,981 SNPs, respectively (Lasky et al., 2012; Forester et al., 2018). A simple RDA model, without correcting for population structure, using the LD-filtered set of 11,421 SNPs and outlier loci were identified in the three significant constrained axes as the SNPs having loadings ±3 SD from the mean score. Gene annotations for the significant candidate SNPs were used to investigate putative adaptive functions, using the Macpha1-annotated vcf.

## Data Availability

Raw sequencing data for the 95 M. phaseolina WGS samples can be accessed at the NCBI SRA accessions SAMN34106355 to SAMN34106449 (http://www.ncbi.nlm.nih.gov/bioproject/953167). The accession number(s) can be found in the article Supplementary Material. The code and data for this study are available at https://github.com/vivianaortizl/Macrophomina_popgen.
