# Supplementary material for: Population genomic analysis reveals geographic structure and climatic diversification for Macrophomina phaseolina isolated from soybean and dry bean across the United States, Puerto Rico, and Colombia
Source: Front Genet. 2023 Jun 7;14:1103969. doi: 10.3389/fgene.2023.1103969 (PMC10282554; doi:10.3389/fgene.2023.1103969)
Supplement: Supplementary file 1 [file Presentation1.zip › Suppl. Figures.DOCX]

Supplementary Material

# Supplementary Figures and Tables

## Supplementary Figures

**Supplementary Figure S1. (A)** Rooted phylogeny reconstructed using the Macpha1 reference genome as outgroup. Maximum-likelihood phylogeny reconstructed using 77,465 high-quality SNPs. Bootstrap support values over 70 are shown at nodes. Bootstrapping converged after 600 replicates. Colored tips represent the genetic cluster for each isolate as defined by principal components analysis. Individual isolate names include ANSI/ISO codes for US states, and Colombia and Puerto Rico municipalities: CA: California, CAU: Cauca, GA: Georgia, IN: Indiana, ISA: Isabela, JD: Juana Diaz, KY: Kentucky, LA: Louisiana, MAG: Magdalena, MI: Michigan, MN: Minnesota, MS: Mississippi, SC: South Carolina, SD: South Dakota, TN: Tennessee, TOL: Tolima, TX: Texas, VAC: Valle del Cauca, WI: Wisconsin. ISO country codes: US: United States, COL: Colombia and PR: Puerto Rico. **(B)** Discriminatory analysis of principal components. Each bar and color indicates the posterior probability membership value per isolate to one of the five genetic clusters.

**
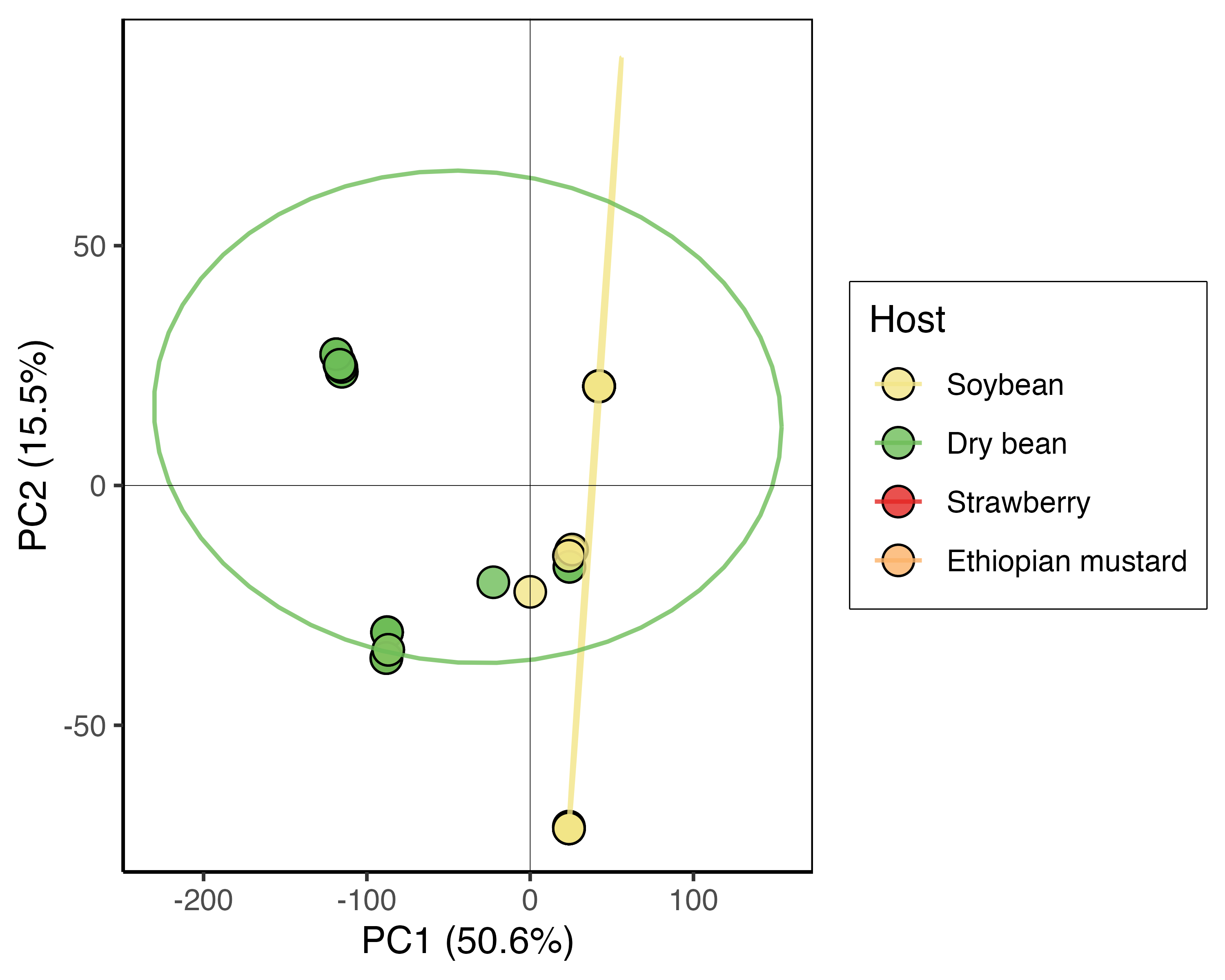
**

**Supplementary Figure S2.** Principal component analysis (PCA) showing isolate host origin. Scatterplot from a principal component analysis based on the two first PCs (the eigenvectors of the 77,465 SNPs) for all isolates. Points are colored by host from which isolates were collected. Overlapping ellipses representing 95% of the isolates from each of the hosts.

**Supplementary Figure S3.** Spatial population structure using conStruct. **(A)** Maps of admixture proportions estimated for *M. phaseolina* across the US, Puerto Rico and Colombia using the spatial conStruct model for K = 2 to K = 4. Pies show mean admixture results for individual isolates within their diameter. **(B)** Cross-validation predictive accuracy values as a function of the number of layers (K = 1-7) for the spatial and nonspatial conStruct models. ﻿**(C)** Layer contributions for K = 2 through 4.


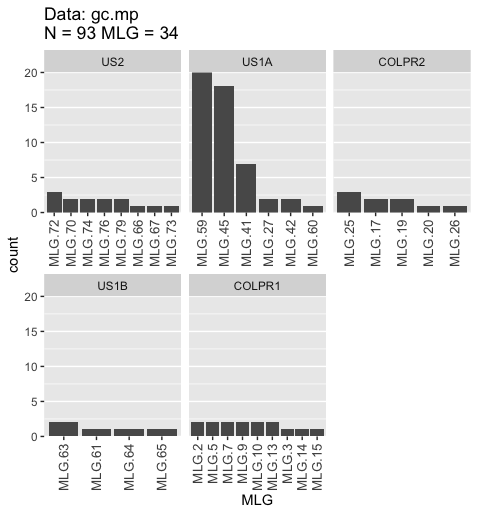

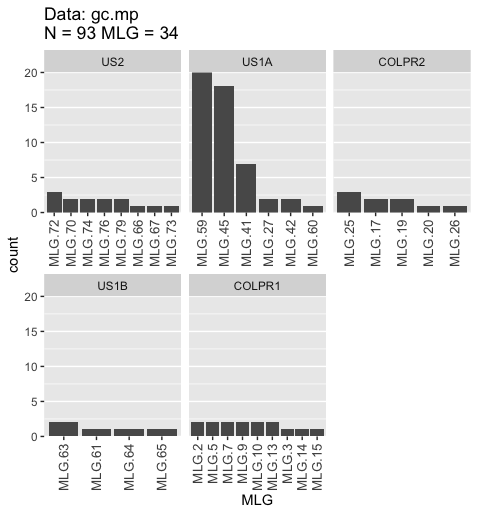

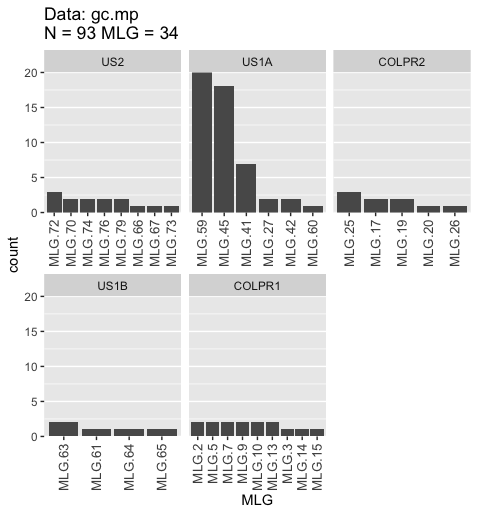

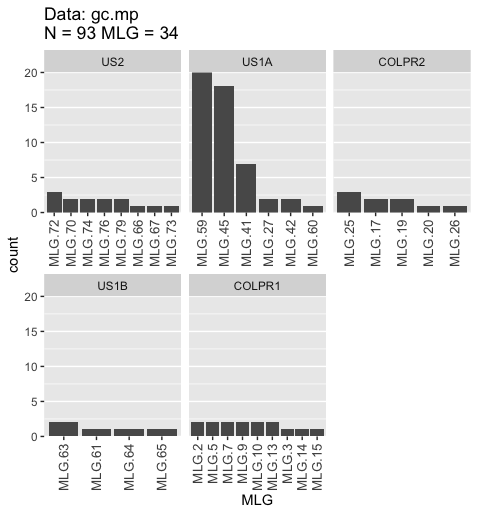

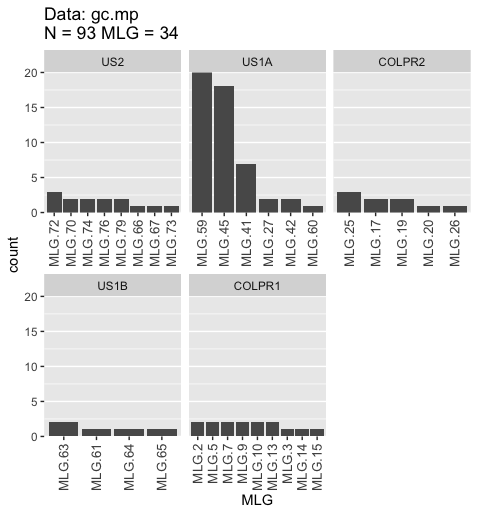

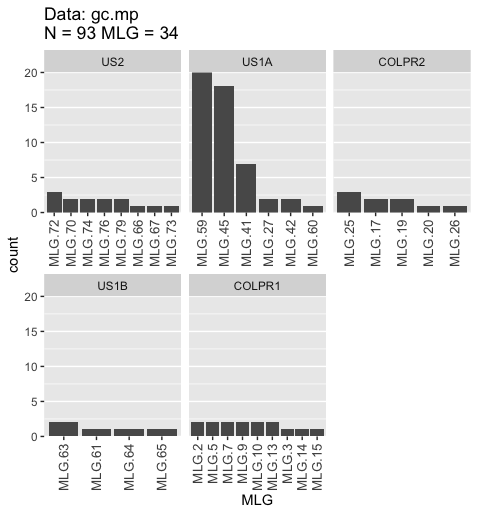

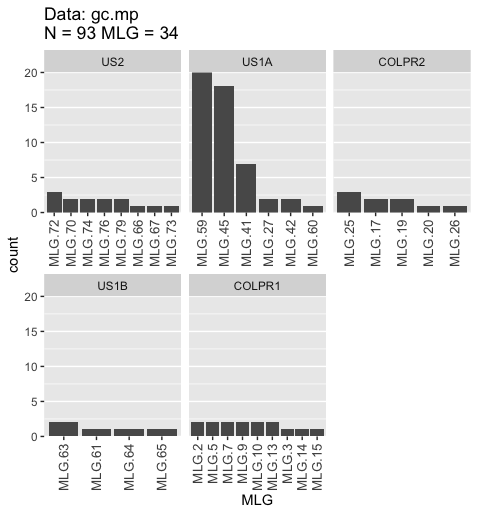


MLL

Count

**Supplementary Figure S4.** MLLs shared among countries. MLL 7: one isolate from Colombia (Mph-5) and one from Puerto Rico (UPR-Mph-JD1) clustering in COLPR1, MLL 17: one isolate from Puerto Rico (UPR-Mph-ISA3) and one from Louisiana (TN501) clustering in COLPR2, and MLL 59: one isolate from Colombia (Mph-49) and 19 isolates from US clustering in US1A . The two MLLs for isolates IN129-4 and Mph40 are not shown.


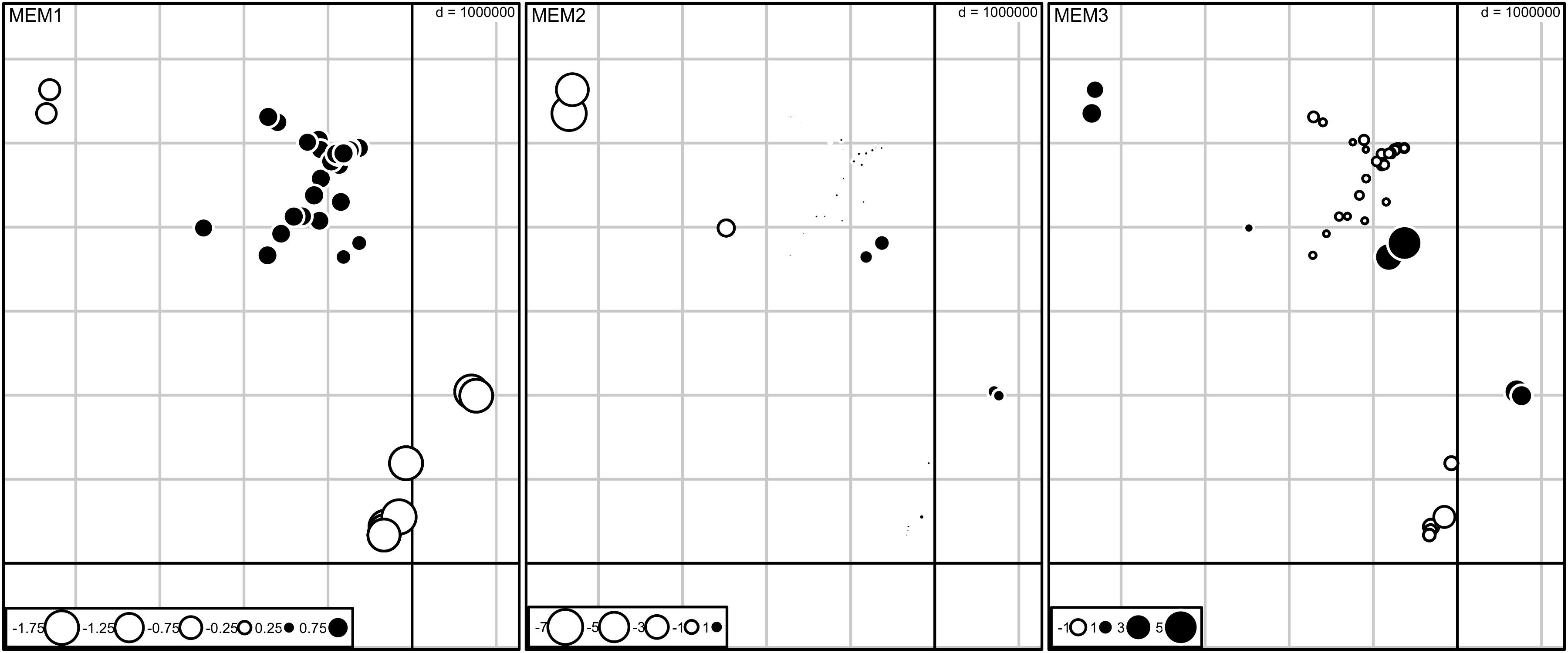


**Supplementary Figure S5.** Spatial structure variables identified using distance-based Moran’s eigenvector maps (dbMEMs 1-3). The variable dbMEM 3 identified as significant using forward-variable selection described broad spatial structure. Color and size of the points correspond to the sign (+ or -) and magnitude of the dbMEM variables, respectively.


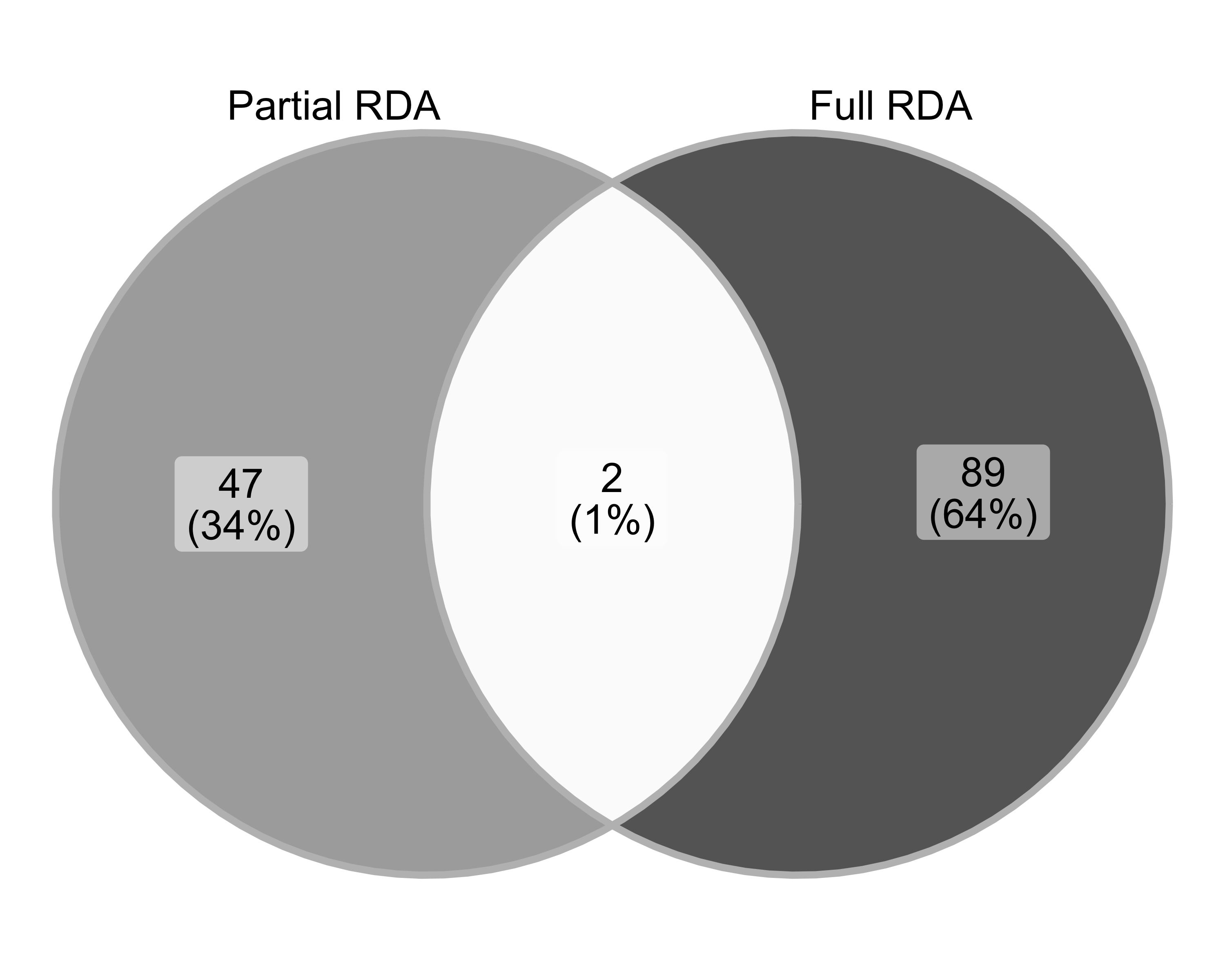


**Supplementary Figure S6.** Venn diagram showing the overlap between outlier loci identified by both partial RDA (constrained on neutral population structure) and full RDA (unconstrained) models using unlinked SNPs (LD-filtered set of 11,421 SNPs).

**Supplementary Figure S7.** Maximum-likelihood phylogeny reconstructed using concatenated sequences of the Internal Transcribed Spacer regions for the nuclear rDNA operon (ITS), part of the Translation Elongation Factor (TEF-1α) gene region, and part of the actin (ACT) gene region.
